# Integrative scATAC-seq and scRNA-seq analyses map thymic *i*NKT cell development and identify *Cbfβ* for its commitment

**DOI:** 10.1038/s41421-023-00547-x

**Published:** 2023-06-20

**Authors:** Jie Wang, Indra Adrianto, Kalpana Subedi, Tingting Liu, Xiaojun Wu, Qijun Yi, Ian Loveless, Congcong Yin, Indrani Datta, Derek B. Sant’Angelo, Mitchell Kronenberg, Li Zhou, Qing-Sheng Mi

**Affiliations:** 1 Center for Cutaneous Biology and Immunology Research, Department of Dermatology, Henry Ford Health, Detroit, MI USA; 2 Immunology Research Program, Henry Ford Cancer Institute, Henry Ford Health, Detroit, MI USA; 3 Center for Bioinformatics, Department of Public Health Sciences, Henry Ford Health, Detroit, MI USA; 4 Department of Medicine, College of Human Medicine, Michigan State University, East Lansing, MI USA; 5 Child Health Institute of New Jersey, Rutgers Robert Wood Johnson Medical School, New Brunswick, NJ USA; 6 La Jolla Institute for Immunology, 9420 Athena Circle, La Jolla, CA USA; 7 Department of Internal Medicine, Henry Ford Health, Detroit, MI USA

**Keywords:** Immunology, Tumour immunology

## Abstract

Unlike conventional αβT cells, invariant natural killer T (*i*NKT) cells complete their terminal differentiation to functional *i*NKT1/2/17 cells in the thymus. However, underlying molecular programs that guide *i*NKT subset differentiation remain unclear. Here, we profiled the transcriptomes of over 17,000 *i*NKT cells and the chromatin accessibility states of over 39,000 *i*NKT cells across four thymic *i*NKT developmental stages using single-cell RNA sequencing (scRNA-seq) and single-cell assay for transposase-accessible chromatin sequencing (scATAC-seq) to define their developmental trajectories. Our study discovered novel features for *i*NKT precursors and different *i*NKT subsets and indicated that *i*NKT2 and *i*NKT17 lineage commitment may occur as early as stage 0 (ST0) by two distinct programs, while *i*NKT1 commitments may occur post ST0. Both *i*NKT1 and *i*NKT2 cells exhibit extensive phenotypic and functional heterogeneity, while *i*NKT17 cells are relatively homogenous. Furthermore, we identified that a novel transcription factor, *Cbfβ*, was highly expressed in *i*NKT progenitor commitment checkpoint, which showed a similar expression trajectory with other known transcription factors for *i*NKT cells development, *Zbtb16* and *Egr2*, and could direct *i*NKT cells fate and drive their effector phenotype differentiation. Conditional deletion of *Cbfβ* blocked early *i*NKT cell development and led to severe impairment of *i*NKT1/2/17 cell differentiation. Overall, our findings uncovered distinct *i*NKT developmental programs as well as their cellular heterogeneity, and identified a novel transcription factor *Cbfβ* as a key regulator for early *i*NKT cell commitment.

## Introduction

Invariant natural killer T (*i*NKT) cells are innate-like T cells that share the characteristics of T cells and NK cells^1,2^ and modulate a broad spectrum of immune responses and diseases^3^. In the mouse thymic cortex, rare CD4^+^CD8^+^ double positive (DP) thymocytes expressing a Vα14-Jα18 T-cell receptor (TCR) chain, preferentially paired with a diverse set of TCR Vβ chains (Vβ8s, Vβ7 or Vβ2 chains), are positively selected by CD1d on DP thymocytes and highly express CD24 (CD24^+^, defined as stage 0 (ST0)). These newly selected *i*NKT cells enter the medulla directed by the expression of *Ccr7*^4^. In the thymic medulla, they sharply downregulate CD24 (CD24^*−*^CD44^*−*^NK1.1^*−*^, defined as stage 1 (ST1)), then upregulate the adhesion molecule CD44 and acquire a memory or activate phenotypes (CD44^hi^NK1.1^*−*^, defined as stage 2 (ST2)). ST2 *i*NKT cells either emigrate to the peripheral organs or remain as long-lived resident cells and mature after acquiring NK1.1 and other NK lineage markers (CD44^hi^ NK1.1^+^, defined as stage 3 (ST3)) in the thymus^1^.

Unlike conventional Th1/2/17 T cells, which differentiate in the peripheral lymphoid tissues upon antigen encounter or specific cytokine treatment, *i*NKT cells acquire their effector function and differentiate into *i*NKT1 (PLZF^lo^T-bet^hi^), *i*NKT2 (PLZF^hi^RORγt^*−*^), and *i*NKT17 (PLZF^int^RORγt^+^) cells prior to thymic export. These *i*NKT subsets have cytokine profiles similar to their Th1/2/17 counterparts, but are less strict. For example, *i*NKT1 cells also produce IL-4, although they mainly produce IFN-γ. Most ST3 *i*NKT cells are either CD4^+^ single positive (CD4^SP^) or CD4^*−*^CD8^*−*^ double negative (DN) cells, while ST2 cells are more diverse, including CD4^SP^ *i*NKT2 cells, DN *i*NKT17 cells, and immature *i*NKT1 cells^5^.

Although advanced studies have been conducted in *i*NKT cells recently, several central questions remain to be answered including: (1) What is the early biological event post *i*NKT positive selection? (2) How do the specific transcription factors coordinated with their chromatin background guide *i*NKT cell sub-lineage commitment and differentiation in the thymus? (3) What is the potential checkpoint for different *i*NKT subset differentiation? (4) Are *i*NKT1, *i*NKT2 and *i*NKT17 cells phenotypically/functionally heterogeneous or homogenous? Recent advances in single-cell assays provide an avenue to explore the transcriptomic and epigenetic heterogeneity of cells at single-cell resolution. Single-cell RNA sequencing (scRNA-seq) can be utilized to assess cell-to-cell variation and has been used to discover rare populations and to infer lineage relationships^6,7^, which offers an unbiased approach to study *i*NKT cell developmental trajectory and heterogeneity. Single-cell assay for transposase-accessible chromatin sequencing (scATAC-seq) offers a similar resolution and provides additional information about gene regulatory processes. Here, we profiled the transcriptomes of over 17,000 *i*NKT cells and the chromatin accessibility states of over 39,000 *i*NKT cells across four thymic *i*NKT developmental stages. By integrating transcriptome and chromatin accessibility profiles, we identified two developmental programs in ST0 that contribute to *i*NKT2 and *i*NKT17 differentiation, while *i*NKT1 commitment occurs in ST1. Both *i*NKT2 and *i*NKT1 cells exhibit extensive heterogeneity, while *i*NKT17 cells are relatively homogenous. We identified a co-transcription factor *Cbfβ* highly expressed in the *i*NKT commitment checkpoint, and conditional deletion of *Cbfβ* in the thymocytes almost totally blocked early *i*NKT cell development and severely impaired *i*NKT1/2/17 cell differentiation. Overall, our study captured *i*NKT cell developmental trajectories, revealed their cellular heterogeneity, and identified *Cbfβ* as a key regulator for early *i*NKT cell commitment.

## Results

### Clustering thymic *i*NKT cells across successive developmental stages by scRNA-seq and scATAC-seq

**Fig. 1:**
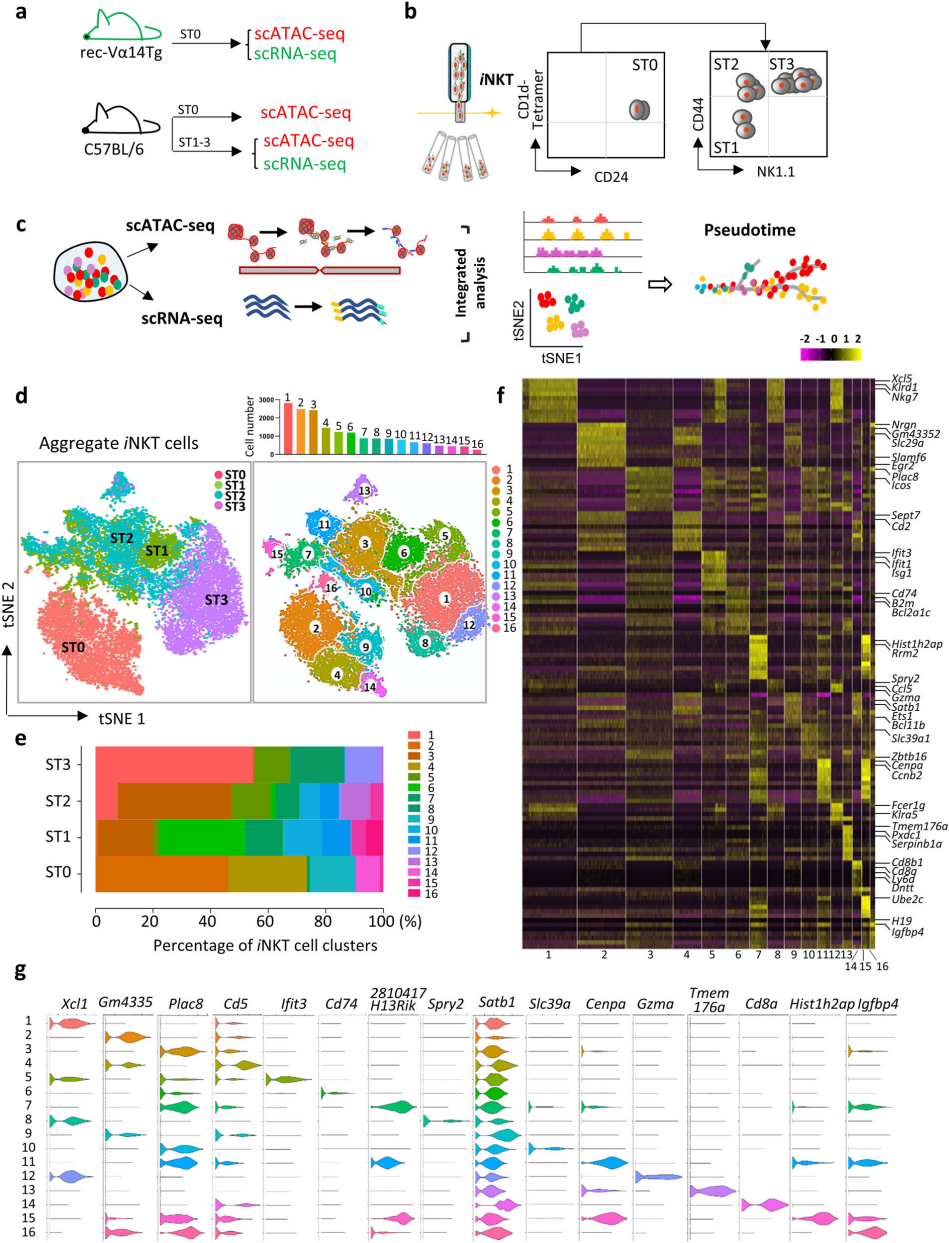
The diversity of mouse thymic *i*NKT cell. **a** *i*NKT cells collected for scATAC-seq and scRNA-seq analysis. **b** Sorting strategy of ST0 (CD24^+^), ST1 (CD24^*−*^CD44^l^^o^NK1.1^*−*^), ST2 (CD24^*−*^CD44^h^^i^NK1.1^*−*^) and ST3 (CD24^*−*^CD44^h^^i^NK1.1^+^) *i*NKT cells. **c** Overview of study design. **d** t-SNE plots from 10X genomics scRNA-seq dataset. Cells from sorted thymic ST0, ST1, ST2 and ST3 *i*NKT cells were pooled. Displaying relationships between individual cells with color-coded on ST0/1/2/3 *i*NKT cells (left); t-SNE plots of data identical to those in the left but color-coded on the different clusters (right). Bar graph represents cell numbers in each cluster (top), *n* = 2. **e** The fractions of sixteen clusters defined in aggregated *i*NKT cells across ST0–3. **f** Heatmap of the top ten DEGs from each cluster derived from (**d**). Each column represents gene expression for an individual cell with color-coded on gene expression profiles. Yellow is upregulated, and purple is downregulated. **g** Violin plots of cluster-defining genes in each cluster derived from (**d**).

To unveil the *i*NKT cell developmental landscape, thymic *i*NKT cells across successive developmental stages were harvested by fluorescence-activated cell sorting (FACS) for scRNA-seq and scATAC-seq assays (Fig. 1a, b). Given the rarity of thymic ST0 (CD24^+^) *i*NKT cells in C57BL/6 mice, we utilized Vα14-Jα18-transgenic mice (also called rec-Vα14Tg) for ST0 *i*NKT cell analysis, which closely mimic the endogenous TCR locus but have abundant ST0 *i*NKT cells^8,9^ (Supplementary Fig. S1a, b). As expected, we found a high similarity in scATAC-seq profile in ST0 *i*NKT cells between the rec-Vα14Tg and C57BL/6 mice (Supplementary Fig. S1c, d). Therefore, we further performed scRNA-seq analyses using rec-Vα14Tg mice for ST0 *i*NKT cells and C57BL/6 mice for ST1 (CD24^*−*^CD44^lo^NK1.1^*−*^), ST2 (CD24^*−*^CD44^hi^NK1.1^*−*^) and ST3 (CD24^*−*^CD44^hi^NK1.1^+^) *i*NKT cells (Fig. 1a–c and Supplementary Fig. S1b).

The scRNA-seq and scATAC-seq libraries were generated using the 10X Genomics platform. After the quality control filtering and excluding cell outliers, a total of 17,944 high-quality single thymic *i*NKT cells with a total of 13,578 expressed genes were retained for the subsequent scRNA-seq analysis. A total of sixteen clusters were identified using the R Seurat package^10,11^ in the aggregated *i*NKT cells (ST0–ST3), from as few as 255 cells to as many as 2817 cells per cluster (Fig. 1d, e). The most differentially expressed genes (DEGs) in each cluster were shown in the heatmap (Fig. 1f) and violin plots (Fig. 1g). Among these clusters, four clusters (C2, C4, C9, and C14) were from ST0, eight clusters were from ST1 (C3, C5, C6, C7, C10, C11, C15, and C16), nine clusters were from ST2 (C1, C3, C5, C6, C7, C10, C11, C13, and C15) and four clusters were from ST3 (C1, C5, C8, and C12) (Fig. 1d and Supplementary Fig. S2a). ST0 *i*NKT cells were clearly separated from the rest of the *i*NKT stages, and the clusters from ST1 and ST2 *i*NKT cells moderately overlapped, whereas *i*NKT clusters from ST3 were closely adjacent to those from ST2 *i*NKT cells (Fig. 1d and Supplementary Fig. S2b). Moreover, the correlation analysis indicated that distinct clusters within the same stage exhibit a relatively similar transcriptomic pattern. For example, the correlation between clusters in ST0 ranged from 0.37 (C2 vs C14) to 0.79 (C2 vs C4), and the correlation between clusters in ST3 ranged from 0.39 (C5 vs C12) to 0.87 (C1 vs C8). As expected, the clusters in different stages did not exhibit a significant correlation, indicating that transcriptomic patterns are greatly distinct in the clusters belonging to different stages (Supplementary Fig. S2c).

**Fig. 2:**
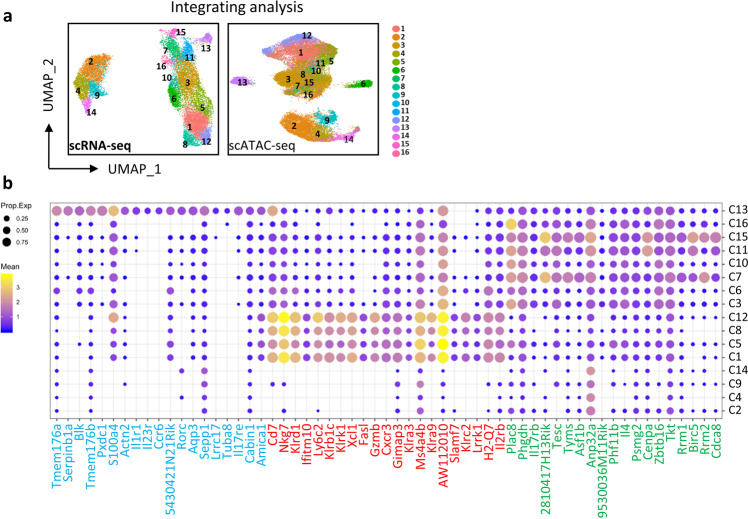
Different clusters assigned into functional subsets. **a** UMAP plots showing integrated analysis of scRNA-seq and scATAC-seq from sorted ST0–3 *i*NKT cells. **b** Bubble plots showing gene expression in individual clusters (C1–C16) from aggregated *i*NKT cells. Gene names labeled in blue are *i*NKT17 signature genes, in red are *i*NKT1 signature genes and in green are *i*NKT2 signature genes. *y* axis shows different clusters identified in (**a**).

Cellular differentiation is accompanied by the expression of genes controlled by cis-regulatory elements, which must be in an open state in order to function properly. We therefore performed scATAC-seq analyses on thymic *i*NKT cells from different developmental stages as those in scRNA-seq analysis and mapped the chromatin accessibility landscape of individual *i*NKT cells using the R Seurat and Signac packages. A total of 39,428 cells were analyzed, with a median of 11,398 fragments per cell mapped to the nuclear genome (Supplementary Fig. S2d). As expected, those sixteen *i*NKT cell clusters were well identifiable after the integration with scRNA-seq data (Fig. 2a). Since scRNA-seq allows us to identify the current cell state as implicated by the transcriptome, we then assigned each of the clusters (C1–C16) into defined *i*NKT1, *i*NKT2, and *i*NKT17 functional subsets based on their signature transcriptomes as previously published^12^ and cytokine transcript expression. We found that C3, C6, C7, C10, C11, C15, and C16 clusters from ST1 and ST2 were categorized into *i*NKT2 subset; C1, C5, C8, and C12 clusters, majorly from ST3 were categorized into *i*NKT1 (Fig. 2b and Supplementary Fig. S2a, e); while a unique *i*NKT C13 from ST2 was assigned into *i*NKT17 (Fig. 2b and Supplementary Fig. S2a, d). As expected, ST0 clusters (C2, C4, C9, and C14) did not stand out in any *i*NKT subsets (Fig. 2b) since they did not exhibit strong effector signatures. Overall, by integrating transcriptomic and epigenetic profiles, we mapped the dynamic transcriptome and chromatin landscapes of thymic *i*NKT cells with sixteen clusters and uncovered the heterogeneity of *i*NKT1 and *i*NKT2 cells and the relative homogeneity of *i*NKT17 cells.

*i*NKT cells proliferate briskly during development, especially at ST1 and ST2, or iNKT2/17 subsets (Supplementary Fig. S3a, b), we wondered whether the cell cycle genes may mask other functionally key genes for *i*NKT cells. To eliminate this confounding factor and unmask the underlying *i*NKT cell heterogeneity, we regressed out cell effects followed by re-clustering of these *i*NKT cells. As shown in Supplementary Fig. S3c–e after removing the effects of the cell cycle on the transcriptome, the clusters observed in ST0 and ST3 are very consistent with our primary data (Fig. 1d). Given that cell proliferation is the nature of ST1 and ST2, and those cells have a very strong capability to expand *i*NKT1, *i*NKT2, and *i*NKT17 subsets, we included those cells for our following analysis.

### Two developmental trajectories in ST0

**Fig. 3:**
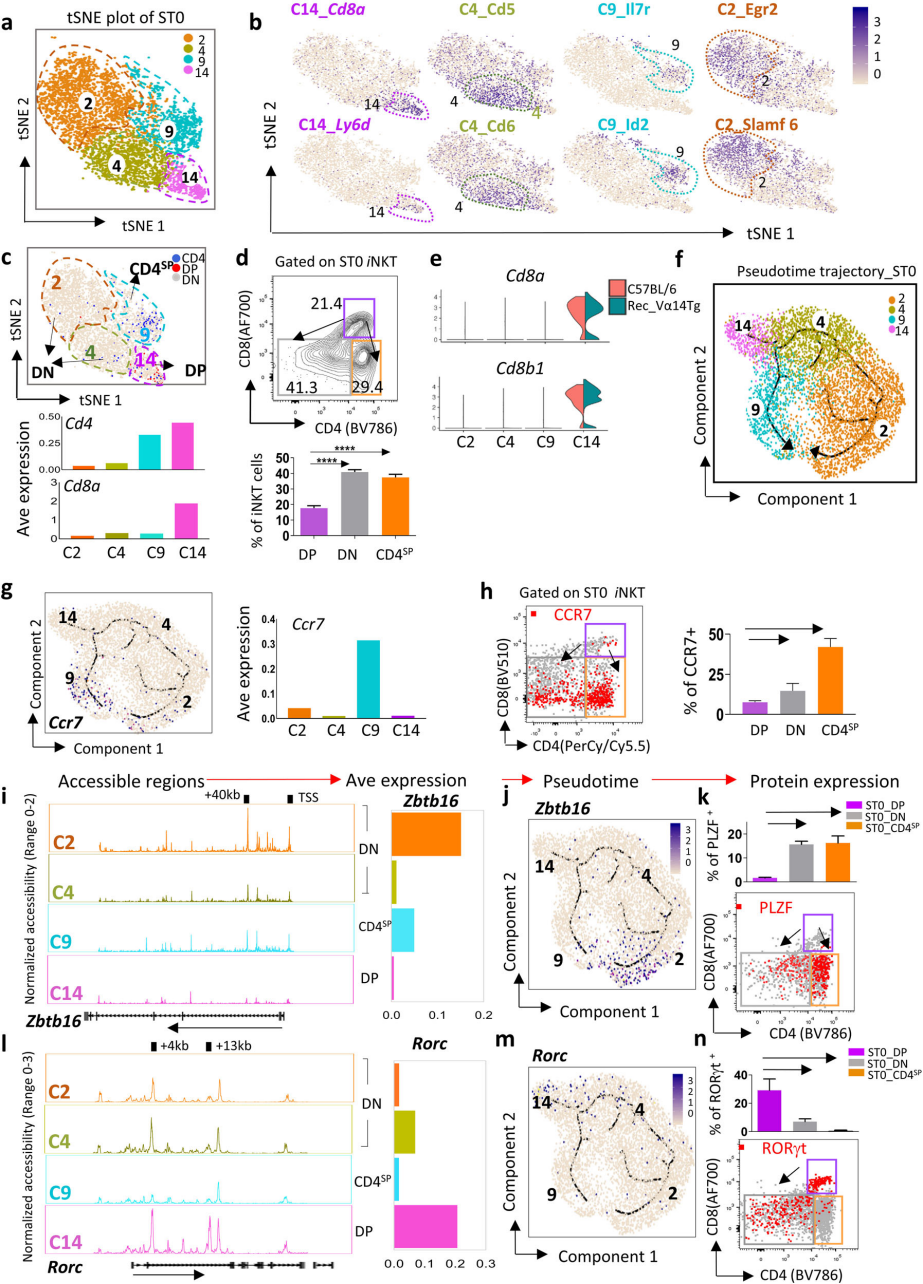
Cellular diversity of *i*NKT cells at ST0. **a** t-SNE plots from scRNA-seq dataset from sorted ST0 *i*NKT precursors (*i*NKTp). **b** Feature plots depicting specific gene expression in each cluster in ST0 *i*NKTp. **c** Feature plot depicting single-cell gene expression of *Cd4*, *Cd8a* and their co-expression (top). Bar graph represents the average (Ave) expression of *Cd4* and *Cd8a* in the clusters (bottom). **d** Representative flow plots of CD4 vs CD8 expression. The bar graph represents means ± SD, *n* = 19. Data represent seven independent experiments (bottom). DP CD4^+^CD8^+^ double positive, DN CD4^*−*^CD8^*−*^ double negative, CD4^SP^ CD4^+^ single positive. **e** Violin plots of *Cd8* expression in each cluster-derived ST0 iNKT cells from C57BL/6 mouse (red) and rec-Vα14Tg mouse (green). **f** The ordering of ST0 *i*NKTp along pseudotime in a state-space defined by Monocle 3. Each color represents a cluster. **g** The same pseudotime plot as in (**f**), feature plots depicting single-cell gene expression trajectory of *Ccr7* in ST0 *i*NKTp development (left). The bar graph represents Ave expression of *Ccr7* (right). **h** Representative flow plots of Ccr7 expression in ST0 *i*NKTp (left). Bar graph presents mean CCR7^+^ *i*NKT ± SD, *n* = 3 (right). **i**, **l** Aggregate scATAC-seq browser tracks for *Zbtb16* (**i**) and *Rorc* (**l**) in ST0 *i*NKTp clusters. The bar graph represents *Zbtb16* (**i**) and *Rorc* (**l**) Ave expression (right). **j**, **m** The same pseudotime plot as in **f**; feature plots depicting single-cell gene expression of *Zbtb16* (**j**) and *Rorc* (**m**) in the ST0 *i*NKTp development trajectory. **k**, **n** Representative flow plots of PLZF (**k**) and RORγt (**n**) expression patterns in ST0 *i*NKTp (bottom). Bar graphs represent mean PLZF^+^ *i*NKT ± SD (**k**) and mean RORγt^+^ *i*NKT ± SD (**n**) (top). *n* = 5, data represent three independent experiments.

*i*NKT cell development in the thymus relies on the pool of around 1000 ST0 *i*NKT precursors (*i*NKTp) located in the thymic cortex^13^. Here, we identified four clusters (C2, C4, C9, and C14) in ST0 (Figs. 1d and 3a) and highlighted the specific signatures for each cluster (Fig. 3b). To further test the robustness of ST0 cells from rec-Vα14Tg mice, we then compared ST0 from rec-Vα14Tg mice with selected CD24^+^ ST0 cells from C57BL/6 mice recently reported by Krovi et al.^14^. After correction of batch effect, merging, and aligning of data from two libraries, ST0 cells from C57BL/6 mice essentially mirrored the ones from rec-Vα14Tg mice with similar clusters (Supplementary Fig. S4a, b). Furthermore, the co-expression of these genes in individual clusters were verified in both rec-Vα14Tg and C57BL/6 mice by flow cytometry (Supplementary Fig. S4c, d). Alternatively, based on CD4 and CD8 expression, these ST0 clusters can be classified into three groups: C14 are CD4^+^CD8^+^ (DP) *i*NKTp; C9 are CD4^+^CD8^*−*^ (CD4^SP^) *i*NKTp, enriched with the regulators of T lymphocyte survival, including *Id2* and *Il7r*; C2 and C4 are CD4^*−*^CD8^*−*^ (DN) *i*NKTp, with C4 highly expressing *Cd5* and *Cd6*, while C2 *i*NKTp abundantly expresses *Egr2* and *Slamf6*, which are essential for *i*NKT cell development via regulation of PLZF expression and TCR signaling strength, respectively^15,16^ (Fig. 3b–d).

Although previous studies claimed that *i*NKT cells are either DN or CD4^SP^ ^17^, a small DP *i*NKTp cluster (C14) was indeed uncovered in ST0 (Fig. 3c–e and Supplementary Fig. S4a–d). To rule out the possibility that C14 DP *i*NKTp might be the contaminated un-signaled DP thymocytes, we analyzed the usage of TCR Vβ8s/β7 in both rec-Vα14Tg and C57BL/6 mice, and found that the Vβ repertoire of DP *i*NKTp was similar to non-DP ST0 *i*NKTp and mature *i*NKT cells. More importantly, the expression of TCR Vβ chains in DP *i*NKTp was significantly higher than that in un-signaled DP thymocytes^18^ (Supplementary Fig. S5a, b). In addition, this cluster also highly expressed recombinase subunits (e.g., *Rag1*, *Rag2*, and *Dntt*) (Supplementary Fig. S6) and early T-cell decision molecule *Ly6d*^19^ (Fig. 3b), indicating that C14 DP *i*NKTp had recently been TCR signaled. Therefore, we assumed that C14 ST0 *i*NKT cells could be the transient *i*NKT precursors from DP thymocytes, which were recently positively selected to *i*NKT cell lineage.

To explore the developmental programs after selection, we applied the Monocle toolkit^20^ in R to organize ST0 *i*NKTp into a pseudotime trajectory. Two potential developmental branches, C14-C2-C4 branch (termed as DP-DN) and C14-C9 branch (DP-CD4^SP^) were identified (Fig. 3f). Both branches eventually meet at the developmental ends with increased expression of chemokine receptor *Ccr7* (Fig. 3g), which is required for ST0 *i*NKTp migration from the thymic cortex to medulla^21^. *Ccr7* expression patterns in DP, DN, and CD4^SP^ *i*NKTp were further confirmed at the protein level by flow cytometry (Fig. 3h). Thus, after selection, DP *i*NKTp downregulate CD8 or both CD4 and CD8 expression to initiate DP-CD4^SP^ or DP-DN developmental programs in the thymic cortex, which eventually migrate into the thymic medulla.

### Functional *i*NKT subset-lineage commitments in ST0

We next asked how these two developmental programs in ST0 contribute to *i*NKT subset-lineage commitments. Although PLZF is annotated as a key *i*NKT2 signature, it is also critical for overall *i*NKT cell development, including *i*NKT1 and *i*NKT17 cells. Thus, we first assessed the expression pattern and chromatin accessibility of *Zbtb16* (encodes PLZF) in ST0 *i*NKTp. We found that the region +40 kb and TSS to *Zbtb16* was not accessible in the DP *i*NKTp (C14) but was accessible in CD4^SP^ (C9) and DN (C2 and C4) *i*NKTp with much higher levels of openness in C2 over C4, which closely resembled the *Zbtb16* expression pattern (Fig. 3i, j). The pseudotime trajectory and flow cytometry further indicated that *Zbtb16* was enriched in both DN and CD4^SP^ *i*NKTp (Fig. 3j, k). Interestingly, PLZF^hi^ *i*NKT cells in CD4^SP^ exhibit stronger TCR signaling strength compared to that in DN *i*NKT cells, indicated by the increased expression levels of PLZF, TCR, Nur77, and Vβ7^22,23^ (Supplementary Fig. S7a). Given that *i*NKT2 differentiation requires the strongest TCR signaling compared to *i*NKT1 and *i*NKT17^24,25^, it is likely that PLZF^hi^ *i*NKTp in CD4^SP^ (C9) prefer to commit into the *i*NKT2 cell lineage.

RORγt is a key transcription factor to regulate *i*NKT17 differentiation but is also highly expressed in un-signaled DP thymocytes and promotes *i*NKT cell selection^26^. Unlike *Zbtb16*, *Rorc* (encode RORγt) at the +4 kb region was accessible in both DP (C14) and DN (C2 and C4) *i*NKTp, but not in the CD4^SP^ (C9) cluster, while +13 kb to *Rorc* was only accessible in DP *i*NKTp (C14). The *Rorc* expression pattern is consistent with its chromatin accessibility status in each cluster (Fig. 3l). *Rorc* was significantly highly expressed in DP *i*NKTp and was gradually downregulated in the DN branch (C4 and C2) but was barely detected in CD4^SP^ (C4) (Fig. 3m), which was further validated by flow cytometry (Fig. 3n). Interestingly, the distinct PLZF^int^RORγt^hi^ *i*NKT population in DN *i*NKTp exhibits a similar phenotype to the mature *i*NKT17 cells (Supplementary Fig. S7b). Thus, it is likely that *i*NKT17 commitment underwent the DP-DN developmental program in ST0 (C14-C4-C2). T-bet (encoded by *Tbx21*) is a key transcription factor to regulate *i*NKT1 cell differentiation^27^. Interestingly, we did not observe obvious *Tbx21* expression in any clusters or open regions near *Tbx21* in ST0 *i*NKTp (data not shown). Overall, our pseudotime-based analysis of developmental trajectories revealed that there might be two potential development programs in ST0, at which *i*NKT cells may initiate their commitment to *i*NKT2 and *i*NKT17 cells that occur as early as ST0 and may initiate *i*NKT1 cells post ST0. However, this hypothesis is still under further investigation.

### *i*NKT cell developmental trajectory

We understand that *i*NKT cells that are presorted from ST0, ST1, ST2, and ST3 may not perfectly present the actual developmental path of their development. To test the robustness of our approach and analyze whether these cells can unveil the model of *i*NKT cell development, we projected cells from different developmental stages on Uniform Manifold Approximation and Projection (UMAP) of a recently published study from unbiased *i*NKT cells thymic population^14^. We found a similar distribution of developmental stages and clusters along the *i*NKT cell development (Supplementary Fig. S8a–c). Furthermore, the gene expressions of cells in each stage for those two datasets are highly correlated (Pearson’s *r* ≥ 0.95; Supplementary Fig. S8d). Thus, we thereafter focused our stage-based *i*NKT cells, which also provided another clue (e.g., stage related) for *i*NKT cell development. We found that *i*NKT cells, especially for *i*NKT1 cells, were following linear “stage” of development; however, iNKT2/17 cells were terminated their differentiation at ST2.

**Fig. 4:**
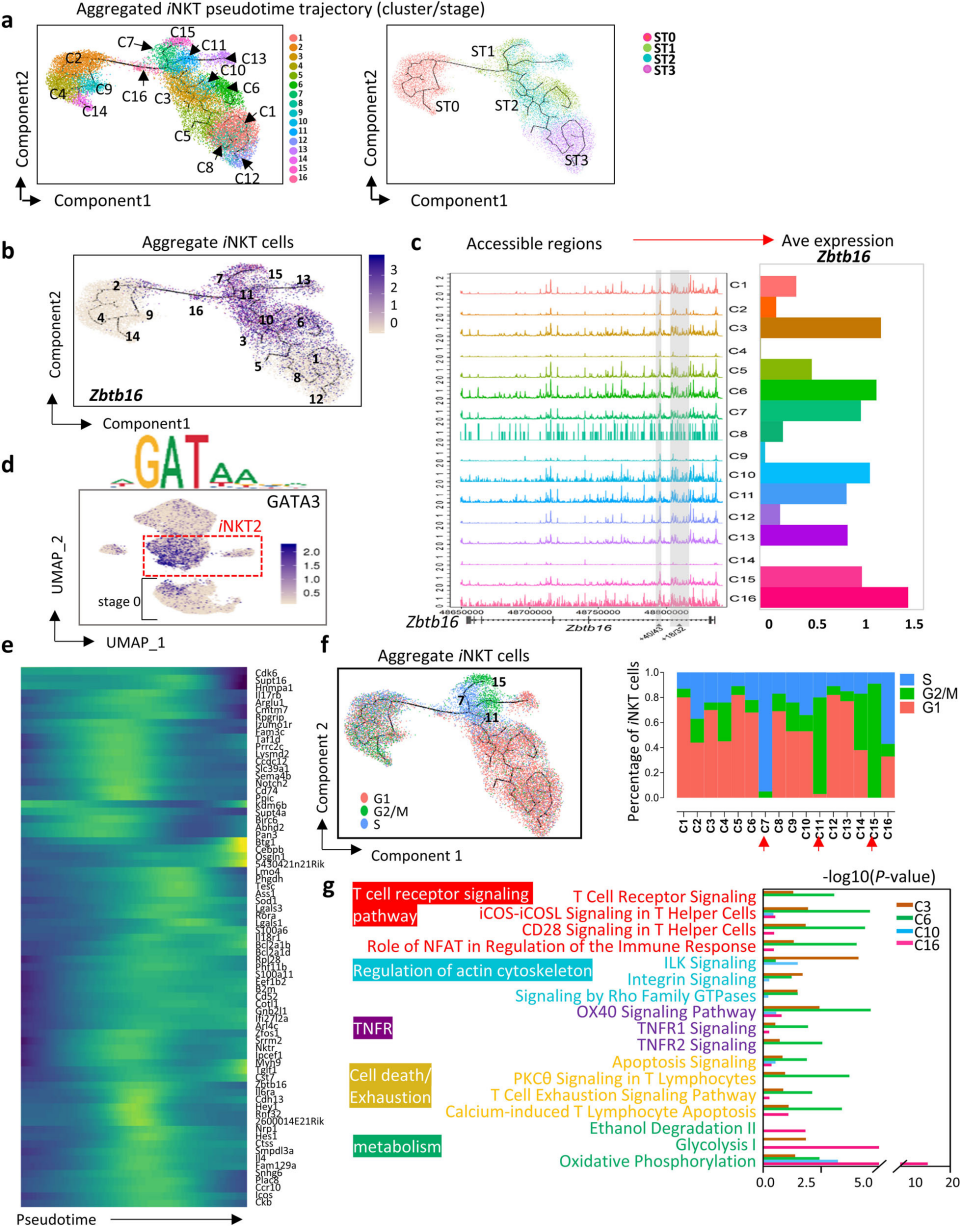
Cellular diversity in the *i*NKT2 cells. **a** The ordering of *i*NKT cells along pseudotime in a state-space defined by Monocle 3. Each color represents an *i*NKT cluster (left) and stage (right). **b** The same pseudotime plot as in (**a**); feature plots depicting single-cell gene expression trajectory of *Zbtb16* in *i*NKT cell development. **c** Aggregate scATAC-seq browser tracks for *Zbtb16* for *i*NKT cell clusters (left). Bar graph represents *Zbtb16* average (Ave) expression in *i*NKT cell clusters (right). **d** UMAP projection colored by the activity of *GATA3*-binding motifs. **e** Heatmap showing pseudotime ordering of most DEGs selected from *i*NKT2 clusters C3, C6, C10, and C16 of scRNA-seq data. **f** Feature plots depicting single-cell gene expression trajectory of G1, G2/M, and S phages in aggregated *i*NKT cell development (left). Bar graph represents fraction of G1, G2/M and S cells in *i*NKT cells clusters (right). **g** DEGs in cluster C3, C6, C10, and C16. Pathway enrichment is expressed as the –log10 (*P* value) adjusted for multiple comparison.

To understand the *i*NKT subset development post ST0, we mapped aggregated thymic *i*NKT cells (ST0–3) into a pseudotime trajectory (Fig. 4a and Supplementary Fig. S9a, b). Two branches stemming from ST0 were merged into a narrow window-C16 at ST1, where *Ccr7* chromatin was much more accessible compared with that of other ST1 clusters (Supplementary Fig. S9c). The dynamic expression pattern of *Zbtb16*, *Tbx21*, and *Rorc* described three *i*NKT subset developmental trajectories, respectively (Supplementary Fig. S10a). *Zbtb16*^hi^ *i*NKT2 (C3, C6, C7, C10, C11, C15, and C16) may initiate their development from both DP-CD4^SP^ (predominately) and DP-DN branches in ST0 and continued throughout ST1 and ST2. Interestingly, C6 cells were terminally ended at ST1 as DN *i*NKT cells (Supplementary Fig. S10a, b); *Tbx21*^+^ *i*NKT1 cells start from CD4^SP^ in ST1 and undergo brisk proliferation transitioned through *i*NKT2 clusters prior to the terminal *i*NKT1 differentiation in ST3. Among the *i*NKT1 pool, C12 as DN *i*NKT cells reach the end of the developmental journey (Supplementary Fig. S10a, b). *Rorc*^+^ *i*NKT17 cells (C13) rooted from the DP-DN branch in ST0 became terminally differentiated at ST2 as DN *i*NKT cells (Supplementary Fig. S10a, b). Collectively, our pseudotime-based analysis of developmental trajectories revealed that both *i*NKT1 and *i*NKT17 cells were at the ends of the trajectory, indicating that they were well differentiated. However, the majority of *i*NKT2 clusters (except C6) were centrally positioned along the *i*NKT cell differentiation axis suggesting a high plasticity in *i*NKT2 cells.

Furthermore, an alternative computational approach, URD^28^, was performed, whereby cluster C14 from ST0 was used as the root point. Consistently, C13 (*i*NKT17) was the first to branch off from the trunk, followed by *i*NKT2 clusters (C10, C6, and C3), and subsequently, the *i*NKT1 clusters (C1, C5, C8, and C12) emerged. Notably, cluster C5 was branched out earlier than C1, C8, and C12 (Supplementary Fig. S10c). Overall, using URD approach, we further validated *i*NKT cell thymic development trajectory.

### Cellular diversity in *i*NKT2 cells

*i*NKT2 cells showed an extensive diversity, including C3, C6, C7, C10, C11, C15, and C16 clusters (Figs. 2b and 4a, b). We first assessed *Zbtb16* chromatin accessibility in integrated *i*NKT cell clusters (C1–C16). In 181 kb of the *Zbtb16* locus, we found highly accessible regions in *i*NKT2, *i*NKT1 and *i*NKT17 clusters, but much weaker regions in ST0 clusters (C2, C4, C9, and C14). *Zbtb16* expression was closely matched to its chromatin accessibility (Fig. 4c). *i*NKT2 clusters and C2 and C9 in ST0 displayed high activity of *GATA3* motif-binding activity, which is critical for *i*NKT2 differentiation^5^ (Fig. 4d). As expected, most signature genes for *i*NKT2 cells gradually increased until reaching their peaks at an intermediate stage, followed by a down-regulation during the terminal differentiation. A few genes, however, including *Btg1*, *Cebpb*, and *Osgin1*, only appeared near the *i*NKT developmental end, which may be related to *i*NKT2 cell terminal events^29–31^ (Fig. 4e).

Previous studies indicated that *i*NKT cells in ST1 and ST2 undergo high proliferation. Cell-cycle pathway enrichment analysis suggested that *i*NKT2 clusters (C7, C11, and C15) exhibited highly proliferative characteristics, which were either in S phase (C7) or G2/M phase (C11 and C15) (Fig. 4f). Ingenuity Pathway Analysis (IPA) indicated that clusters C3, C6, C10, and C16 are functionally different. C16 is a transient phase of *i*NKT cells from ST0 to ST1, and these cells sharply elevated expression of genes associated with both glycolysis and oxidative phosphorylation (Fig. 4g), suggesting their increased energy demands^32^. C6 terminated at ST1, and these cells enriched with genes in TCR signals, co-stimulation signaling, cytoskeleton, TNFR, and cell death and exhaustion pathways (Fig. 4g). This cluster highly expresses *Cd74* (Supplementary Fig. S11a), a gene associated with class II major histocompatibility complex and related to T cell–T cell interaction^33^. Consistently, flow cytometry analysis further confirmed that the majority of CD74^+^ *i*NKT cells were in ST1 (Supplementary Fig. S11b). However, the functional profiles of C6 are still under investigation. Taken together, *i*NKT2 exhibit a great cellular diversity.

### Heterogeneity of *i*NKT1 cells

**Fig. 5:**
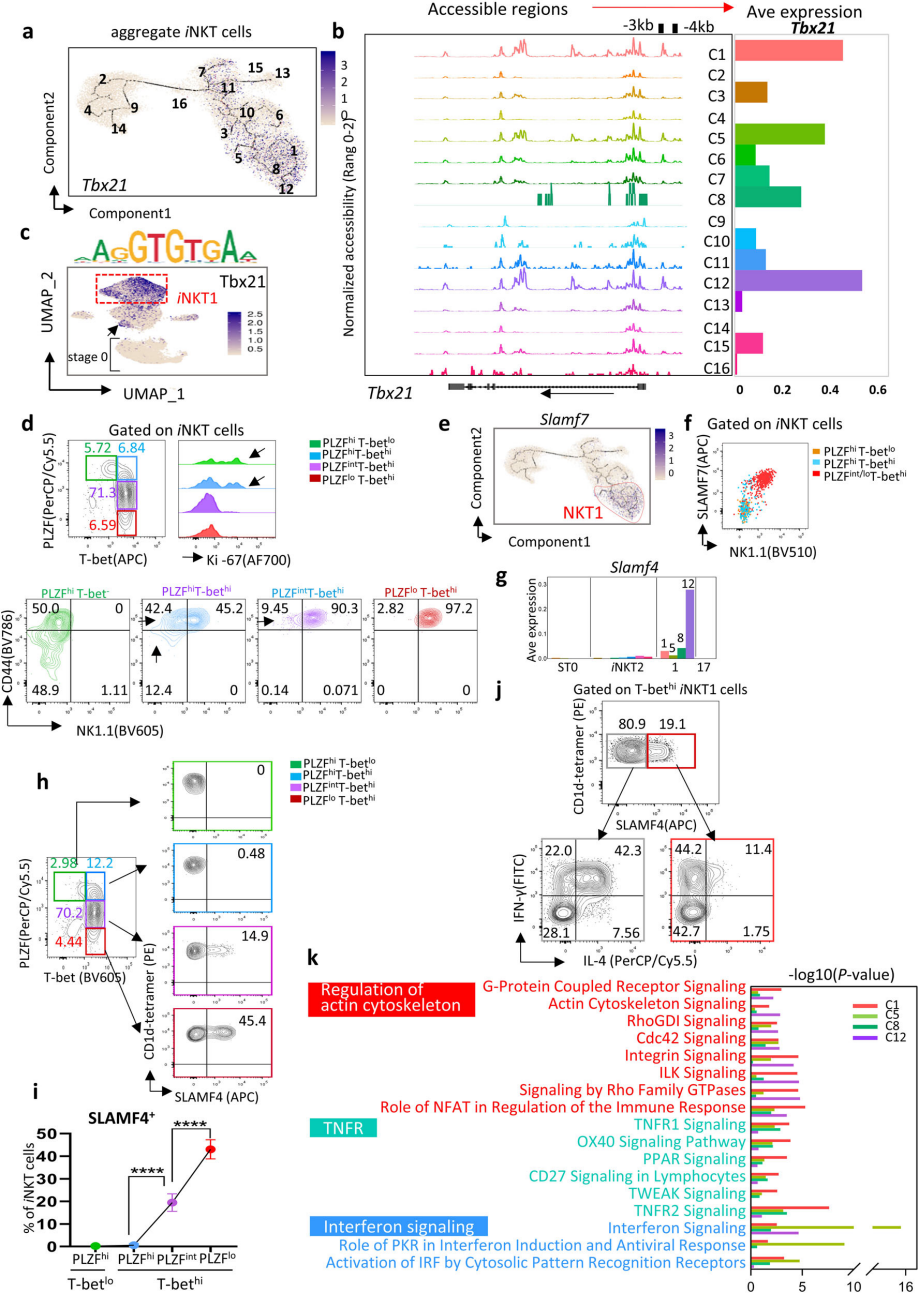
Extensive cellular heterogeneity of *i*NKT1 cells. **a** The same pseudotime plot as in Fig. 4a; feature plots depicting single-cell gene expression trajectory of *Tbx21* in *i*NKT cell development. **b** Aggregate scATAC-seq browser tracks for *Tbx21* for *i*NKT cell clusters (left). Bar graph represents *Tbx21* average (Ave) expression in *i*NKT cell clusters (right). **c** UMAP projection colored by the activity of *Tbx21*-binding motif. **d** Representative flow plots of PLZF vs T-bet gated on *i*NKT cells, *i*NKT2 (PLZF^hi^T-bet^*−*^) in green and different *i*NKT1 (T-bet^hi^) cells, marked by PLZF high (PLZF^hi^T-bet^hi^) in blue, PLZF medium (PLZF^int^T-bet^hi^) in purple, and PLZF low (PLZF^lo^T-bet^hi^) in red (top left). Histogram showing Ki-67 expression in the indicated *i*NKT subsets derived from the right. Representative flow plots of CD44 vs NK1.1 expression in the indicated *i*NKT sub-population derived from top (bottom). **e** The same pseudotime plot as in Fig. 4a; feature plots depicting single-cell gene expression trajectory of *Slamf7* in *i*NKT cell development. **f** Representative flow plots of SLAMF7 vs NK1.1 expression in *i*NKT cells. PLZF^hi^T-bet^lo^ in orange, PLZF^hi^T-bet^hi^ in blue, PLZF^int/lo^T-bet^hi^ in red. **g** Bar graph represents *Slamf4* Ave expression in *i*NKT cell clusters. **h** Representative flow plots of SLAMF4 expression in *i*NKT2 (PLZF^hi^T-bet^*−*^) in green and different *i*NKT1 (T-bet^hi^) cells, marked by PLZF high (PLZF^hi^T-bet^hi^) in blue, PLZF medium (PLZF^int^T-bet^hi^) in purple, and PLZF low (PLZF^lo^T-bet^hi^) in red. **i** Dot graph represents mean SLAMF4^+^ ± SD. *n* = 9, Data represent three independent experiments, and were analyzed by a two-sided paired *t* test, ****P < 0.0001. **j** Representative flow plots of IL-4 vs IFN-γ production in SLAMF4^*−*^ (left) and SLAMF4^+^ (right) *i*NKT1 cells post PMA/Ionomycin stimulation for 4 h, *n* = 5, Data represent three independent experiments. **k** DEGs in clusters C1, C5, C8, and C12. Pathway enrichment is expressed as the –log10 (*P* value) adjusted for multiple comparison.

*i*NKT1 cell differentiation was controlled by transcription factor *Tbx21* and assigned to four clusters (C1, C5, C8, and C12) (Figs. 2b and 5a). Within 17.2 kb of the *Tbx21* locus, the promoter regions –3 kb and –4 kb were highly accessible in C1, C5, and C12, but were less accessible in C8 (Fig. 5b). The same regions were also accessible in hyper-proliferative C7, C11, and C15 *i*NKT2 clusters in ST1 and ST2. Importantly, *Tbx21*-binding motif activity also occurred in these clusters, but not in ST0 clusters (Fig. 5c). Flow cytometry analysis further confirmed that a small fraction of PLZF^hi^*i*NKT2 cells express T-bet in ST1 and ST2, with considerable proliferation ability as measured by Ki-67 (Fig. 5d). Therefore, it is possible that *i*NKT1 progenitors might hide in these so-called proliferative PLZF^hi^T-bet^hi^ *i*NKT2 cells.

Pseudotime analysis showed that most *i*NKT1 signatures were not highly expressed until reaching the end of the *i*NKT development continuum (Supplementary Fig. S12a). We found that a novel signature, signaling lymphocytic activation molecule family member 7 (*Slamf7*), is enriched in *i*NKT1 cell clusters (Fig. 5e). Flow cytometry further confirmed this based on the strong correlation between SLAM7 and NK1.1 expression in PLZF^lo^T-bet^hi^ *i*NKT1 cells (Fig. 5f). NK cell-related signature *Slamf4* further distinguished terminal ended C12 from other *i*NKT1 cell clusters. SLAMF4^+^ *i*NKT cells (C12) were mainly DN, and gradually sprouted out from PLZF^lo^T-bet^hi^ *i*NKT1 cells (Fig. 5g–i and Supplementary Fig. S12b, c). SLAMF4^+^ *i*NKT1 cells mainly secreted IFN-γ with less IL-4, like “classical *i*NKT1 cells”, while the majority of SLAMF4^*−*^ *i*NKT1 cells secreted both IL-4 and IFN-γ upon stimulation (Fig. 5j). Furthermore, a small population of SLAMF4^+^PLZF^lo^T-bet^hi^ *i*NKT1 cells also expressed soluble cytotoxic mediator *Gzma*, identified as a cytotoxic *i*NKT1 population (Supplementary Fig. S12d–f). However, these cytotoxic GZMA^*+*^SLAMF4^+^ *i*NKT1 cells were barely detected in the peripheral organs (Supplementary Fig. S12g). C5 cells were enriched with *Ifit1* and *Ifit3*, which are involved in the interferon signaling pathway^34^ (Fig. 5k and Supplementary Fig. S12h). Overall, *i*NKT1 cells start their journey from as currently defined “*i*NKT2 cells” at ST1, and gradually complete their differentiation and turn into SLAMF4^+^ *i*NKT1 cells at the end, as classical IFN-γ-secreting *i*NKT1 cells.

### *i*NKT17 cells exhibit limited heterogeneity

**Fig. 6:**
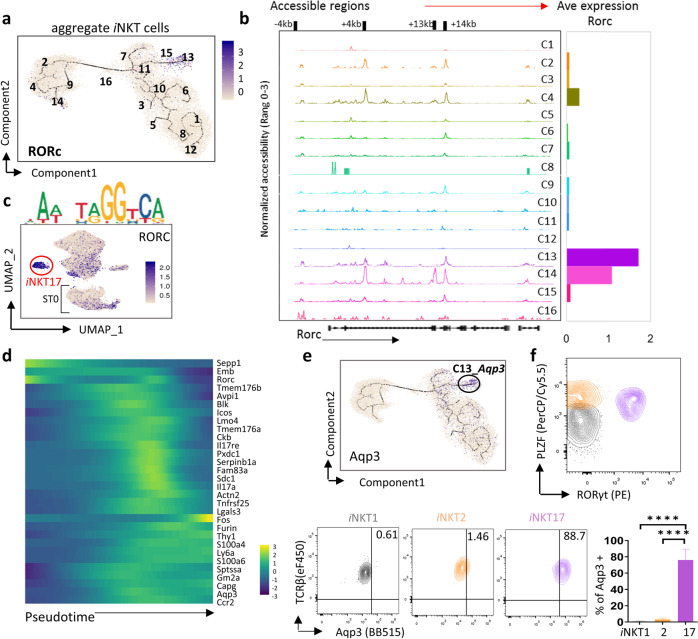
*i*NKT17 cells exhibit limited heterogeneity. **a** The same pseudotime plot as in Fig. 4a; feature plots depicting single-cell gene expression trajectory of *Rorc* in *i*NKT cell development. **b** Aggregate scATAC-seq browser tracks for *Rorc* for *i*NKT cell clusters (left). Bar graph represents *Rorc* average (Ave) expression in *i*NKT cell clusters (right). **c** UMAP projection colored by the activity of *RORC*-binding motif. **d** Heatmap showing pseudotime ordering of top 30 genes in cluster 13 of scRNA-seq data. **e** The same pseudotime plot as in Fig. 4a; feature plots depicting single-cell gene expression trajectory of *Aqp3* in *i*NKT cell development. **f** Representative flow plots of Aqp3 expression in *i*NKT1 (PLZF^*−*^RORγt^*−*^, gray), *i*NKT2 (PLZF^hi^ RORγt^*−*^, orange) and *i*NKT17 (PLZF^int^RORγt^+^, purple). Bar graph represents mean Aqp3^+^ *i*NKT ± SD, *n* = 8. Data represent three independent experiments, and were analyzed by a two-sided paired *t* test, ****P < 0.0001.

C13 was assigned as *i*NKT17 cells, which was segregated distinctively from other clusters (Figs. 2b and 6a). We observed that +4 kb and +14 kb regions of *Rorc* were accessible in ST0 clusters (C2, C4, and C14) and mature *i*NKT17 cluster (C13) (Fig. 6b), and binding motifs of *RORC* were activated in *i*NKT17 cluster as well as in ST0 *i*NKTp (Fig. 6c). These data suggested that *i*NKT cells may start their *i*NKT17 commitment at ST0 and complete their differentiation at C13. At mRNA level, *Rorc* was highly expressed in C14 in ST0, which was consistent with the chromatin accessibility at *Rorc*, but was gradually downregulated in C4 and C2, and re-upregulated in C13, indicating that other transcription factor(s) may target the open *Rocr* sites and manipulate *Rorc* expression during *i*NKT17 differentiation.

To trace the developmental trajectory of *i*NKT17 cells, we further checked *i*NKT17 signature genes in an ordered *i*NKT cell trajectory and found that few *i*NKT17 signatures including *Rorc* were initially expressed in the early *i*NKTp, before they reached the mature *i*NKT17. However, the majority of *i*NKT17-related signatures were barely expressed in *i*NKTp (Fig. 6d). We further found that a novel signature aquaporin-3 (Aqp3) was specifically expressed in thymic *i*NKT17 (PLZF^int^RORγt^+^) cells (Fig. 6e, f). Overall, *i*NKT cells likely initiate *i*NKT17 commitment at ST0 and these *i*NKT17 cells exhibits limited heterogeneity.

### *Cbfβ* regulates *i*NKT cell early commitment

**Fig. 7:**
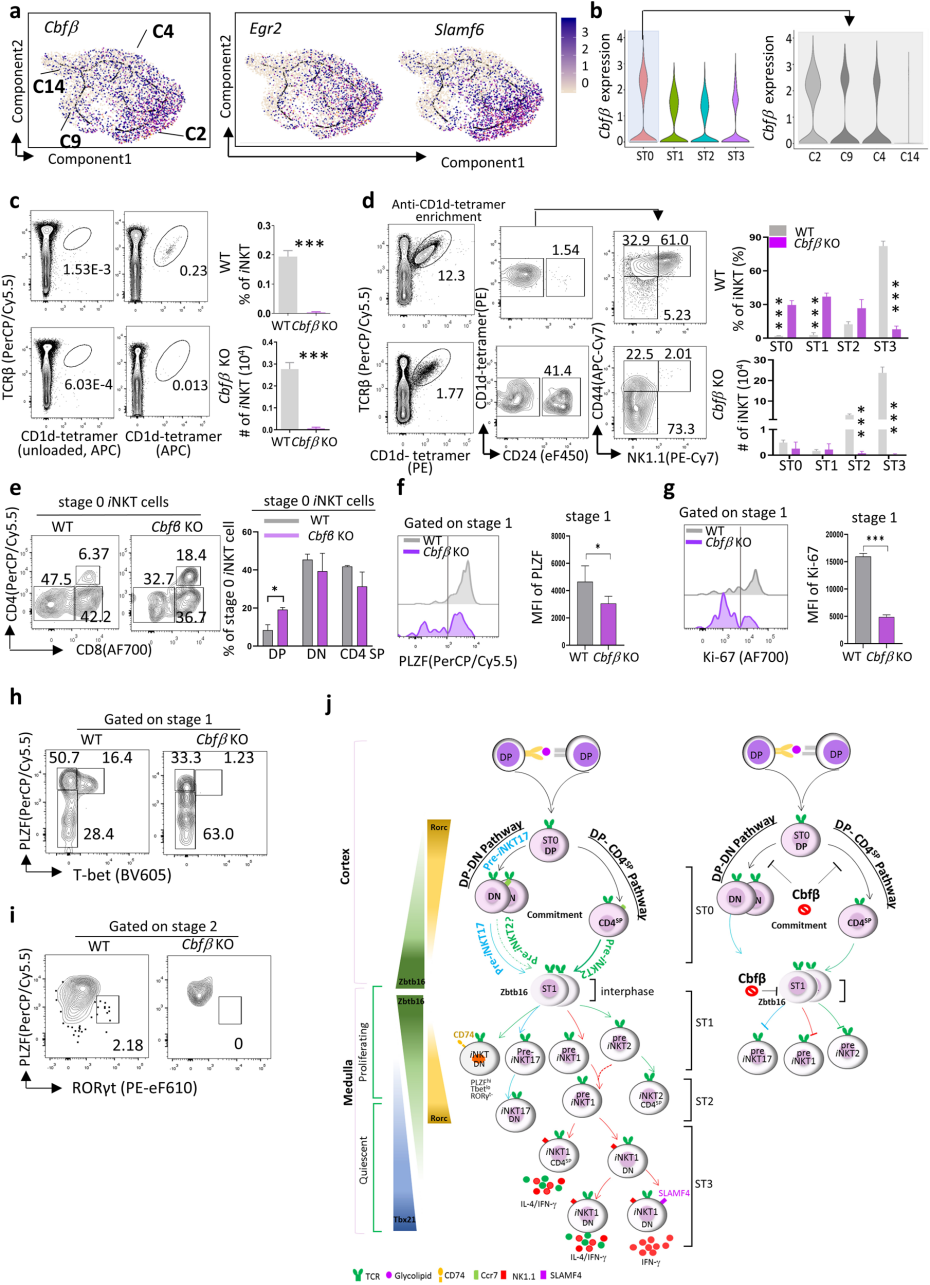
*Cbfβ* regulates *i*NKT cell early commitment. **a** *i*NKT ST0 pseudotime plot; feature plots depicting single-cell gene expression trajectory of *Cbfβ, Egr2*, and *Slamf6* at ST0 *i*NKT cells development (Fig. 3f). **b** Violin plots of *Cbfβ* expression in different stages of *i*NKT cells, and *Cbfβ* expression in different clusters (C2, C9, C4, and C14) from ST0 *i*NKT cells. **c** Representative flow plots of *i*NKT cells from *Cbfβ* KO and WT mice (left). Bar graphs represent mean ± SD of *i*NKT cell frequency and *i*NKT cell number (right), *n* = 5 for *Cbfβ* KO and WT controls. Data represent three independent experiments. **d** Representative flow plots of different stages of *i*NKT (after anti-CD1d-tetramer enrichment) from *Cbfβ* KO and WT mice (left). Bar graphs represent mean ± SD of *i*NKT cell frequency and *i*NKT cell number in *Cbfβ* KO and WT controls (right). **e** Representative flow plots of CD8 and CD4 expression in ST0 *i*NKT cells from *Cbfβ* KO and WT controls (left). Bar graph represents means ± SD of DP, DN, and CD4 SP ± SD (right). WT controls, *n* = 6; *Cbfβ* KO, *n* = 7. Data represent three independent experiments, data were analyzed by a two-sided unpaired *t* test, *P < 0.05. **f**, **g** Histogram showing PLZF expression (**f**) and Ki-67 expression (**g**) in ST1 *i*NKT cells from *Cbfβ* KO and WT mice. Bar graph represents means ± SD of indicated *i*NKT population. WT, *n* = 4. *Cbfβ* KO, *n* = 6. Data represent two independent experiments, and were analyzed by a two-sided unpaired *t* test, *P < 0.05, ***P < 0.001. **h** Representative flow plots of PLZF vs T-bet expression in ST1 *i*NKT cells from *Cbfβ* KO and WT mice. **i** Representative flow plots of PLZF vs RORγt expression in ST2 *i*NKT cells from *Cbfβ* KO and WT mice. **j** The speculated schematic model of mouse *i*NKT cell developmental trajectory (left) and the role of *Cbfβ* in *i*NKT cell development (right).

*Egr2* and *Slamf6* were reported to control early *i*NKT cell development by modulating *Zbtb16* expression and TCR in ST0^15,16,35^. Here, we identified a novel co-transcription factor, *Cbfβ*, which showed a similar expression pattern with *Egr2* and *Slamf6*, and exhibited a great enrichment at ST0 of *i*NKT cells, specifically in the DP-DN branch C2 (Fig. 7a, b).

*Cbfβ*-encoded CBFB is a non-DNA-binding regulatory subunit that allosterically enhances the sequence-specific DNA-binding capacity of RUNX (RUNX1, RUNX2, and RUNX3), and therefore modulates the transcription of their target genes. Here we observed that *Runx1* shows a similar expression trajectory as *Cbfβ* in thymic *i*NKT cells (Supplementary Fig. S13a) even though the expression level is relative lower. More interestingly, consistent with the high expression of *Cbfb* in C2, we observed a greater RUNX1-binding activity in C2, compared with in other clusters in ST0 (Supplementary Fig. S13b). In mammals, two RNA splice variants, *Cbfβ1* and *Cbfβ2*, are generated from a single *Cbfβ* gene, and each variant has distinct amino acid sequences at the C terminus^36^. To determine if *Cbfβ* regulates *i*NKT development, we examined *i*NKT cell development in thymic-specific *Cbfβ* knockout mice (CD4^Cre^*Cbfβ* ^*f/f*^, *Cbfβ* KO), in which both *Cbfβ1 and Cbfβ2* were deleted. *Cbfβ* deletion was further confirmed in protein level and mRNA level in different T-cell subsets (Supplementary Fig. S14a, b). We observed that conventional αβT cells with *Cbfβ* deletion phenotypically mimicked those in RUNX3 KO mice^37^, as *Cbfβ* KO mice displayed abnormalities in CD4 expression, impairment of CD8 T cells maturation in thymus, and a large proportion of DP cells in peripheral (Supplementary Fig. S14c, d). Interestingly, deletion of *Cbfβ* led to a severe reduction in the frequency and absolute number of thymic *i*NKT cells (Fig. 7c), as well as in the peripheral lymphoid organs (Supplementary Fig. S14e), which phenotypically mimicked *i*NKT cells in *Runx1* deletion mice^38^, but not *Runx3* KO mice. This phenomenal explained a similar expression pattern of *Cbfβ* and *Runx* during *i*NKT cell development and higher binding activity of RUNX1 at C2 ST0 (Supplementary Fig. S13a, b). A more comprehensive analysis revealed a selective and significant reduction in the frequency and absolute number of ST2 and ST3 *i*NKT cells in *Cbfβ* KO mice. The frequencies of ST0 and ST1 *i*NKT cells were significantly increased in *Cbfβ* KO mice, but the absolute numbers were comparable between *Cbfβ* KO and WT controls (Fig. 7d). Of interest, DP *i*NKTp were increased in ST0 *i*NKT cells in *Cbfβ* KO mice (Fig. 7e), suggesting that the deletion of *Cbfβ* partially blocks DP *i*NKTp conversion to either CD4^SP^ or DN lineages. We assume that *Cbfβ* may affect *i*NKT cells selection at DP. *Egr2* showed a similar expression pattern as *Cbfβ* in ST0 (Fig. 7a), and is critical for *i*NKT lineage commitment at DP^35^, we therefore detected Egr2 expression at DP stage. As shown in Supplementary Fig. S14f, Egr2 expression was significantly reduced in DP thymocytes from *Cbfβ* KO mice, indicating that Egr2 may involve in *Cbfβ-*mediated iNKT cell development.

ST1 *i*NKT cell undergoes briskly proliferation and contains the progenitors of *i*NKT subsets with high *Zbtb16*-encoded PLZF expression. Here we observed that PLZF expression at ST1 was significantly downregulated in *Cbfβ* KO *i*NKT cells (Fig. 7f). Similar phenomenon was also observed on a proliferating marker, Ki-67 (Fig. 7g). These data suggested that *Cbfβ*-deficient *i*NKT cells entered a relative quiescent status and were unable to normally upregulate PLZF expression at ST1. Furthermore, the remnant PLZF^+^ *i*NKT cells in ST1 fail to co-express T-bet to initiate *i*NKT1 differentiation and to co-express RORγt to initiate *i*NKT17 differentiation (Fig. 7h, i and Supplementary Fig. S14g). Bone marrow chimera transfer experiments showed that there was a sever defect on *i*NKT cell development from the *Cbfβ-*deficient donors (Supplementary Fig. S15a, b), suggesting that the defective *i*NKT cell development in *Cbfβ* KO mice was cell-intrinsic. Overall, our study suggests that *Cbfβ* serves as a key regulator to control early *i*NKT cell development at ST0, *i*NKT cell differentiation at ST1/2, and final maturation at ST3 (Fig. 7j).

## Discussion

*i*NKT cell development was previously considered as a linear model with four successive stages (ST0–ST3). However, recent studies have indicated that thymic *i*NKT cell development is a complex cellular differentiation process and the linear developmental model does not apply to all *i*NKT subsets^13^. A new functional classification of three terminally differentiated subsets, *i*NKT1/2/17, was proposed based on transcription factor expression and cytokine production patterns^12,26^. Very recently, Thomas Baranek et al. performed scRNA-seq on thymic whole *i*NKT cells pool and proposed a model for *i*NKT cell effector differentiation in which *i*NKT1 and *i*NKT17 subsets derive from *i*NKT2. Moreover, *i*NKT1 subset arises linearly and sequentially from *i*NKT2 cells^39^. This study yielded strong evidence for *i*NKT cell development and differentiation models. However, are *i*NKT2 cells the earliest cells pool for iNKT1/17 lineages commitment? If no, how early can their progenitor be identified? To this end, we applied both scRNA-seq and scATAC-seq analyses of thymic *i*NKT cells and mapped the developmental landscape of terminal *i*NKT1/2/17 subsets on *i*NKT development. We did single-cell analysis based on “stages” of development, given the following reasons: (1) There might be some key features of *i*NKT cells at the very early stage, which only take up about 0.5% of total *i*NKT cells in mouse thymus, and sorting whole *i*NKT cells as a pool may hide key features in this rare population. Thus, sorting *i*NKT cells based on the stages would make sure that enough *i*NKT cells at earlier stages were included for further clustering analysis; in this case, we included a total of 7591 ST0 cells for scRNA-seq and scATAC-seq analysis; (2) By pooling different stages of *i*NKT cells, we were also able to assign *i*NKT1/2/17 into different clusters from different developmental stages based on published signature markers. And this could allow us to trace *i*NKT1/2/17 differentiation trajectory in different stages and identify their progenitors. With the integration of single-cell transcriptome and chromatin accessibility analysis, we found that *i*NKT2 and *i*NKT17 lineage commitment may occur in ST0 by two distinct programs, and *i*NKT1 lineage commitment may occur post ST0. Finally, we identified that transcription factor *Cbfβ* plays a key role in *i*NKT cell commitment.

Previous studies suggested that *i*NKT cells arise from a common progenitor designated as PLZF^hi^CD24^+^ in ST0, which further differentiate into *i*NKT1/2/17 subsets^5,40^. However, it is still unclear how and at which specific time window their effector programs unfold during their development. In our current study, we found that ST0 *i*NKTp exhibit extreme heterogeneity, suggesting that *i*NKTp may be destined to the specific subset lineages in ST0. A rare DP *i*NKT cell population that has been ignored previously and hidden in ST0 is likely the earliest *i*NKTp post-positive selection. These DP *i*NKTp then differentiate to either CD4^SP^ or DN *i*NKTp in ST0, and form two distinct developmental programs, DP-CD4^SP^ and DP-DN. The two developmental branches differed in many aspects including distinct transcriptomes, diversity of TCR Vβ usage, and TCR signaling strength. Interestingly, *Zbtb16* upregulation only occurred at the end of DP-CD4^SP^ and DP-DN developmental branches. *Zbtb16*^+^ *i*NKTp in the CD4^SP^ path showed a stronger TCR signaling strength and increased Vβ7 usage compared to that in DP-DN *i*NKTp, suggesting that the *i*NKTp in the DP-CD4^SP^ development program prefer to differentiate into *i*NKT2 cells. However, *Rorc* chromatin accessibility and gene expression are highly enriched in DP *i*NKTp and the DP-DN developmental branch, supporting the notion that *i*NKTp in the DP-DN branch prefer to be differentiated into *i*NKT17 cells. We did not observe any open-chromatin regions near *Tbx21* and its gene expression in ST0 *i*NKTp, but *Tbx21* chromatin accessibility and its gene expression occurred as early as ST1. These findings highly suggest that *i*NKT2 and *i*NKT17 are pre-determined at ST0; however, *i*NKT1 are progressively determined at ST1.

Although three subsets of *i*NKT cells were defined post ST0 based on transcription factor and cytokine secretion profiles, *i*NKT1 and *i*NKT2 cells exhibit extensive phenotypic and functional heterogeneity, while *i*NKT17 cells are relatively homogenous. The trajectory of *i*NKT development showed that *i*NKT17 and *i*NKT1 branches were both sprouted from the developmental tree. However, the *i*NKT2 cells, which were highly proliferative and heterogeneous, were located at the center of developmental trunk. Interestingly, we found that both *Tbx21* and *Rorc* chromatins were also accessible in these proliferative *i*NKT2 clusters, indicating that these proliferative clusters hidden in so-called “*i*NKT2 cells” might contribute to early *i*NKT1/2/17 expansion and differentiation. These results further supported a recent notion that currently defined *i*NKT2 cells may contain mature *i*NKT2, transitioning *i*NKT17, and transitioning *i*NKT1^41^. Aqp3 belongs to a family of highly conserved transmembrane channels that transport water and, in some cases, small solutes such as glycerol. Recent studies indicated that Aqp3 is expressed on T cells and regulates their trafficking in skin and lung immune reactions^42,43^. T-cell migration toward chemokines is dependent on Aqp3-mediated hydrogen peroxide (H_2_O_2_) uptake. Here, our fate-mapping identified that Aqp3, as a new biomarker, is specifically expressed in *i*NKT17 cells. It will be very interesting to further investigate Aqp3’s functions in *i*NKT17 cells, especially for their trafficking to peripheral organs.

*i*NKT cells express multiple Slam family receptors, but only *Slamf1*, *Slamf5* and *Slamf6* are highly expressed in ST0 *i*NKTp (data not shown), which have been reported to be required for *i*NKT cell development^15^. Here, we found that *Slamf7*, as a new marker, is specifically expressed in *i*NKT1 cells. Most importantly, we found that *Slamf4* was enriched in terminal DN *i*NKT1cluster (C12). Given the dynamic transcription factor expression and cytokine production profiles, the *Slamf4*^*+*^ *i*NKT1 cells are likely the terminal differentiated *i*NKT1 cells as “classical *i*NKT1 cells” that highly secrete IFN-γ. Interestingly, the few *Slamf4*^*+*^ *i*NKT1 cells might receive attention as novel cancer therapeutic targets.

*i*NKT10 cells are a novel *i*NKT subset identified upon stimulation of the strong agonist αGalcer, which are characterized by transcription factor *Nfil3* (also term *E4bp4*) and IL-10 production^44^. Previous study suggested that the appearance of *i*NKT10 after αGalcer stimulation could be the result of selective expansion of a rare population of pre-existing *i*NKT10 cells or some non-*i*NKT10 cells converting into the *i*NKT10 phenotype. It is, however, important to point out that *Nfil3* was surprisingly found to be enriched in thymic *i*NKT17 cluster and a small fraction of *i*NKT1 cells. However, IL-10 expression was undetectable in thymic *i*NKT cells (data not shown). It is still unknown whether these *Nfil3*^*+*^ *i*NKT cells can be converted into *i*NKT10 cells following αGalcer stimulation. More comprehensive study is still ongoing.

Collectively, our study generated a comprehensive atlas of thymic *i*NKT cells and their developmental trajectory, providing a valuable resource for future studies of *i*NKT cell biology. We have also uncovered *Cbfβ* as a novel regulator of early *i*NKT cell development (Fig. 7j).

## Materials and methods

### Mice

C57BL/6 were purchased from Jackson Laboratory (Bar Harbor, ME). Rec-Vα14Tg TCR transgenic mice were generated in Dr. Derek Sant’Angelo laboratory^9^, which require *Rag*-mediated recombination to produce a functional TCR (Supplementary Fig. S1a). These mice have increased numbers of *i*NKT cells, as compared with C57BL/6 mouse. Mice carrying a conditional floxed allele of *Cbfβ* (*Cbfβ*^fl/fl^) were previously described^45^ and provided by Dan R. Littman (New York University, New York, NK). Mice were backcrossed to the C57BL/6 background for 7 generations and then mated to C57BL/6 mice carrying the *Cd4* enhancer/promoter/silence *Cre* allele (obtained from The Jackson Laboratory), to generate *CD4*^*Cre*^*Cbfβ* ^fl/fl^ conditional knockout mice (*Cbfβ* KO). The full list of mouse strains used can be found in Supplementary Table S1. 5-week-old, sex-matched mice were utilized in this study. All studies, protocol, and mouse handling were approved by the Institutional Animal Care and Use Committee.

### Flow cytometry gating strategy and antibodies

Single-cell suspensions were washed twice with FACS staining buffer (1× PBS, 2% FBS) and incubated with Fc block (clone 2.4G2). Cells were stained with anti-mouse PBS57-loaded and -unloaded CD1d-tetramer (provided by the NIH Tetramer Core Facility), the following fluorescence conjugated antibodies were used: anti-TCRβ (H57-597), anti-CD24 (M1/69), anti-CD44 (IM7), anti-NK1.1 (PK136), anti-TCR Vβ8.1/8.2 (KJ16-133.18), anti-TCR Vβ8.3 (1B3.3), anti-TCR Vβ7 (TR310), anti-Nur77 (12.14), anti-Ly6d (49-H4), anti-CD5 (53-7.3), anti-CD6 (IM348), anti-Egr2 (erongr2), anti-ID2 (ILCID2), anti-IL7R (A7R34), anti-Cbfβ, anti-Aqp3, anti-CD8 (53-6.7), anti-CD4 (GK1.5), anti-RORγt (B2D), anti-PLZF (Mags.21F7), anti-T-bet (eBio4B10 (4B10)), anti-Slamf7 (520914), anti-Slamf4 (eBio244F4), anti-CD45.1(A20), and anti-CD45.2 (104). Cell surface staining was performed with staining buffer; intranuclear staining for anti-Aqp3, anti-PLZF, anti-T-bet, and anti-RORγt were performed with eBioscience Fixation/permeabilization buffer. The flow cytometry assay was performed through BD FACSCelesta and data were analyzed using FlowJo V10.2 software. Gating strategy: after gating on lymphocyte, doublets were excluded by using forward scatter (FSC), and side scatter (SSC); mouse *i*NKT cells were further identified as TCRβ^+^CD1d-tetramer^+^. The full list of antibodies, reagent, and software used can be found in Supplementary Table S1.

### Bone marrow chimeras transfer experiment

To generate bone marrow chimeras, 7- to 8-week-old C57BL/6. SJL (B6.SJL) recipient mice were lethally irradiated initially with 9.5 Gy with a dose rate of 2.5 Gy per min. Quality assurance of the radiation exposure was performed using multiple dosimetry endpoints including an electrometer, micro-TLDs, and Gafchromic film. Donor bone marrows were harvested from age- and sex-matched SJL (CD45.1^+^) and *Cbfβ* KO (CD45.2^+^) or WT control mice (CD45.2^+^). After erythrocyte lysis, mature T cells (CD3^+^) were depleted by biotin-conjugated anti-mouse CD3 (BD Biosciences) mAbs and anti-biotin magnetic beads (BD Biosciences) from bone marrows of each donor, using Magni Sort^TM^. Over 90% of mature T-cell depletion was confirmed by flow cytometry. CD45.1^+^ SJL and CD45.2^+^ *Cbfβ* KO or CD45.2^+^ WT littermate control bone marrows were mixed at a 1:1 ratio, and 1 × 10^7^ cells per mouse (in a volume of 100 μL) were then injected into the irradiated recipients via tail vein. The chimeras were analyzed 8 weeks after reconstitution.

### Mouse *i*NKT cell enrichment and sorting

Mouse thymi were harvested from 5-week-old C57BL/6 mice and rec-Vα14Tg mice. For the enrichment of *i*NKT cells, thymocytes were stained with APC-conjugated CD1d-tetramer, bound to anti-APC MicroBeads (Miltenyi Biotec), and enriched with an autoMACS Pro Separator (Miltenyi Biotec) using POSSEL_S program. ST1 (CD24^*−*^CD44^lo^NK1.1^*−*^), ST2 (CD24^-^CD44^hi^NK1.1^*−*^), and ST3 (CD24^*−*^CD44^hi^NK1.1^+^) *i*NKT cells were further sorted from C57BL/6 mouse thymus, and ST0 *i*NKT cells (CD24^+^) were sorted from both C57BL/6 and rec-Vα14Tg mice using FACSAria II Usage.

### Quantitative RT-PCR analysis

Total RNA was isolated by Sigma-Aldrich kit and was quantified by a NanoDrop ND-1000 spectrophotometer. The *A*_260_/*A*_280_ ratio was > 1.9 for all the samples. cDNA was prepared by using a cDNA synthesis kit (Sigma-Aldrich) following the manufacturer’s instruction. Primer pairs for *Cbfβ* are: forward (F): 5′-GGTTAGGAGTCATTGTGATCA-3′; reverse (R): 5′-CCTCCTCATTCTAACAGGAATC-3′. The PCR amplification was carried out on the Applied Biosystem 7900 Real-time PCR system; relative quantification using ΔCT values in the cells from *Cbfβ* KO vs WT control was carried out, and fold changes were calculated.

### Western blot analysis

The T-cell protein lysates were prepared using a RIPA buffer containing protease inhibitors. Next, the T-cell protein lysates were subjected to 12% SDS-PAGE analysis with 10 µg of lysate loaded per lane. The anti-Cbfβ rabbit polyclonal antibody was obtained from ThermoFisher Scientific. The anti-GAPDH monoclonal antibody was purchased from Sigma-Aldrich.

### ScRNA-seq library generation

Two biological repeats for each stage of scRNA-seq libraries were generated using the 10X Genomics Chromium Single Cell 3′ Reagent Kit (v2 Chemistry) and Chromium Single Cell Controller as previously described^46^. Briefly, ~5000 cells were loaded into each reaction for gel bead-in-emulsion (GEM) generation and cell barcoding. Reverse transcription of the GEM (GEM-RT) was performed in a Thermocycler (Veriti™ 96-Well Fast Thermal Cycler, Applied Biosynthesis; 53 °C 45 min, 85 °C 5 min, 4 °C hold). cDNA amplification was performed after GEM-RT cleanup with Dynabeads MyOne Silane (ThermoFisher Scientific) with the same Thermocycler (98 °C 3 min; 98 °C 15 s, 67 °C 20 s, 72 °C 1 min, repeat 12 cycles; 72 °C 1 min, 4 °C hold). Amplified cDNA was cleaned up with SPRIselect Reagent Kit (Beckman Coulter) followed by library construction procedure, including fragmentation, end repaired, adaptor ligation, and library amplification. A Bioanalyzer (Agilent) was used for library quality control. cDNA libraries were sequenced on an Illumina HiSeq 4000 using paired-end flow cells (Read 1, 26 cycles, i7 index 8 cycles; Read 2: 110 cycles) by the University of Michigan DNA Sequencing Core facility following the manufacturer’s protocol.

### ScRNA-seq data analysis

Sequenced reads from scRNA-seq libraries were demultiplexed, aligned to the mm10 mouse reference, barcode processed, and Unique Molecular Identifier (UMI) counted using the 10X Genomics Cell Ranger (v2.0.1) pipeline^46^. Estimated number of cells captured per sample were between 1296–3290 with 34,258–95,264 mean reads per cell, 764–1892 median genes per cell, and 1266–6412 median UMI counts per cell. A total of 19,044 cells with 4238 UMI counts/cell in average were selected via Cell Ranger for further analysis for all of six samples. Datasets were subsequently analyzed using the R Seurat package^10,11^. Principle Component Analysis (PCA) was employed to analyze combined samples. Quality control metrics employed are as follows. We employed two strategies to identify potential doublets. Firstly, cells expressing both Xist and Y chromosome genes (Kdm5d, Eif2s3y, Gm29650, Uty, and Ddx3y) were excluded from the dataset. Secondly, cells expressing uncharacteristically high numbers of genes (> 4000) were excluded. Low-quality cells were excluded based on a low number of genes detected (< 300) and/or having high mitochondrial genetic content (> 5%). Additionally, uninteresting sources of variation within the data were removed. Genes removed include ribosomal structural proteins (as identified by gene ontology term GO:0003735 and the Ribosomal Protein Gene (RPG) database^47^), non-coding rRNAs, Hbb, and genes not expressed in ≥ 3 cells. A total of 13,578 genes in 17,944 cells passed these quality control measures.

A global-scaling normalization method “LogNormalize” in Seurat was employed to normalize gene expression measurements of each cell by the total expression, multiplied by a factor of 10,000, followed by log-transformation. Highly variable genes in each data analysis were identified, and the intersecting top 1000 genes in each dataset were used for clustering and downstream analyses. Datasets underwent scaling and regressing on the number of detected molecules per cell and the percentage mitochondrial gene content (pct.mito). The number of principal components (PCs) used to cluster cells was determined by manual inspection of the scree plot. After identifying the number of PCs to be included for downstream analyses (25 PCs), a graph-based clustering approach implemented in Seurat was used to iteratively cluster cells into groups, based on similarities of those components among cells. The t-Distributed Stochastic Neighbor Embedding (t-SNE) method was utilized to visualize resulting clusters. To assess the effects of cell cycle heterogeneity, cell cycle phase scores (G2/M and S phases) were calculated based on canonical markers and used to regress out the data^48^. The *FindAllMarkers* function in Seurat was then implemented to identify DEGs between clusters with a fold-change of > 2 and a Bonferroni adjustment of *P* value < 0.05 as a statistical significance threshold. To determine if DEGs belong to identifiable groups, pathway analysis was carried out IPA (Qiagen Bioinformatics, Redwood City, CA).

### Comparison analysis between our *i*NKT scRNA-seq dataset with the one from ref. ^14^

To compare our scRNA-seq dataset with recently published scRNA-seq data from unbiased *i*NKT cell thymic populations by Krovi et al.^14^ (GEO accession: GSE152786), an integration analysis implemented in the R Seurat package was employed^49^. After performing a normalization using regularized negative binomial regression (the *SCTransform* function) for each dataset, a set of cell pairs or “anchors” between datasets that are assumed to have a similar biological state was identified (the *SelectIntegrationFeatures, PrepSCTIntegration* and *FindIntegrationAnchors* functions) using a canonical correlation analysis (CCA) and mutual nearest neighbors (MNNs). These ‘anchors’ are utilized as the reference to merge the datasets using the *IntegrateData* function. The *i*NKT stages and cluster labels from our scRNA-seq dataset are then projected onto the Krovi et al.’s dataset via the *FindTransferAnchors* and *TransferData* functions.

### ScATAC-seq library generation

FACS-sorted stages 0, 1, 2, and 3 *i*NKT cells from C57BL/6 and rec-Vα14Tg (ST0) mouse thymi were used to generate Chromium Single Cell ATAC Libraries following the recommended protocol (10X Genomics, Pleasanton, CA) as previously described^50^. Briefly, after isolating, washing, and counting of nuclei suspensions, the recovered nuclei were combined with the ATAC buffer and enzyme to create a transposition mix in a tube and incubated at 37 °C for 60 min. A master mix that includes the barcoding reagent, reducing agent, and barcoding enzyme was then added to the same tube. This solution was loaded into the 10X Genomics Chromium Single Cell Controller instrument along with the vortexed scATAC gel beads and partition oil. The resulting GEMs were amplified in a Thermocycler (Veriti™ 96-Well Fast Thermal Cycler, Applied Biosynthesis; 72 °C for 5 min, 98 °C for 30 s, cycled 12×: 98 °C for 10 s, 59 °C for 30 s, and 72 °C for 1 min). After cleaning up using the Recovery Agent, Dynabeads MyOne Silane, and SPRIselect Reagent, the resulting accessible DNA fragments underwent the barcoded and indexed sequencing library construction. These scATAC-seq libraries were sequenced on the Illumina NovaSeq 6000 using paired-end flow cells (50 bp Read 1, 8 bp i7 index, 16 bp i5 index, and 49 bp Read 2) by the University of Michigan DNA Sequencing Core facility following the manufacturer’s protocol.

### ScATAC-seq data processing

Sequenced reads from scATAC-seq libraries were processed using the 10X Genomics Cell Ranger ATAC (v1.0.1). Specifically, raw sequencing reads were demultiplexed and converted into FASTQ files using the *cellranger-atac mkfastq* pipeline. Then, the sequencing reads underwent the *cellranger-atac count* pipeline consisting of read filtering, alignment to the reference genome using STAR, barcode counting, identification of transposase cut sites and accessible chromatin peaks, cell calling, generation of count matrices for chromatin peaks and transcription factors. Estimated number of cells captured per sample were between 2536–11,015 with 11,401–16,343 median fragments per cell and 55,349–87,603 total peaks detected. Then, the output files from multiple samples were combined using the *cellranger aggr* command with read depth normalization to generate a single feature-barcode matrix for all the data. A total of 39,428 cells with a median of 11,398 fragments per cell were selected for further analysis for all samples using the R Seurat and Signac packages (https://satijalab.org/signac/). Cells with sufficient numbers of fragments in peaks (> 3000 and < 100,000), a high percentage (> 20%) of fragments in peaks, a blacklist ratio (a ratio of reads in the regions associated with artificial signals based on the ENCODE Project) of < 0.02 and a nucleosome signal strength of < 10 were kept. A frequency-inverse document frequency (TF-IDF) normalization was performed to normalize sequencing depth differences across cells and provide higher weights to rare peaks using the *RunTFIDF* function. For dimensionality reduction, a singular value decomposition (SVD) was performed using the *RunSVD* function after selecting the top 95% of peaks via the *FindTopFeatures(*object, min.cutoff = “q5”) function. Non-linear dimensionality reductions using t-SNE (the *RunTSNE* function on the first 30 dimensions) and UMAP^51^, the *RunUMAP* function on the first 30 dimensions) methods for visualization, constructing a Shared Nearest Neighbor (SNN) graph (the *FindNeighbors* function), cell clustering (the *FindClusters* function with a resolution of 0.6), and cluster differential accessibility (the *FindAllMarkers* function) were conducted subsequently. To quantify the chromatin accessibility at the gene levels, the fragments for each cell that intersect the gene and promoter (2000 bp upstream) regions were counted. The gene coordinates were obtained from the Ensembl Mus musculus v79 (EnsDb.Mmusculus. v79 R package). To perform motif analysis, the presence of known transcription factor-binding motifs based on the JASPAR 2018 database was scanned within the DNA sequence of each peak. Hence, over-representative motifs in a specific cluster can be identified using the *FindMotifs* function.

### Integrated analysis of scRNA-seq and scATAC-Seq

To perform an integrative analysis of scATAC-seq and scRNA-seq data, an anchoring approach as implemented in the R Seurat and Signac packages was employed^49^. Briefly, a set of cell pairs or “anchors” between datasets assumed to share a similar biological state was first identified (the *FindTransferAnchors* function) using a CCA and MNNs. These “anchors” are then used as the reference to integrate the datasets and the cluster labels from scRNA-seq data can be projected onto the scATAC-seq data via the *TransferData* function. Hence, single-cell measurements across different modalities can be evaluated.

### Pseudotime analysis

Pseudotime analysis of scRNA-seq data was performed using Monocle^52^ (v. 2.99.3) to infer the trajectories of cells during their development. Monocle utilizes the reversed graph embedding technique to construct the trajectories based on the cell similarity in gene expression profiles. Specifically, the size factors for each cell and dispersion function for the genes in the data were first calculated. Next, the data were projected onto the top principal components using the *preprocessCDS* function with parameters as follows: (method = “PCA"', norm_method = “log”, num_dim = 30) and the UMAP dimensionality reduction using the *reduceDimension* function (method = ‘UMAP’, metric = “cosine”, min_dist = 0.75, n_neighbors = 100, num_dim = 30) were applied based on the top 2000 of highly variable genes. After partitioning the cells into groups using the *partitionCells* function, Monocle organizes cells into trajectories based on the concept of reversed graph embedding called SimplePPT, which learns a tree-like trajectory, using the *learnGraph* function (RGE_method = ‘SimplePPT’, prune_graph = F, close_loop = T).

### IPA

To infer the functionality of subset-specific gene signatures and open-chromatin regions, pathway analysis using the IPA (Qiagen Bioinformatics, Redwood City, CA) was performed. This analysis provides information on the potential functions of novel cell subsets that can be validated in mechanistic studies.

### Statistical analysis

For comparison between groups, statistical analysis was performed by unpaired *t* test with GraphPad Prism 8.0.

## Supplementary information

Supplementary Figures

Supplementary Table S1

## Data Availability

ScRNA-seq and scATAC-seq data that support the findings of this study have been deposited in the NCBI Gene Expression Omnibus (GEO; http://www.ncbi.nlm.nih.gov/geo/) with the accession numbers GSE130184 and GSE141825, respectively (Supplementary Table S1). All relevant data are available from the authors upon reasonable request.
