# Supplementary material for: Integrative scATAC-seq and scRNA-seq analyses map thymic iNKT cell development and identify Cbfβ for its commitment
Source: Cell Discov. 2023 Jun 20;9:61. doi: 10.1038/s41421-023-00547-x (PMC10279728; doi:10.1038/s41421-023-00547-x)
Supplement: Supplementary file 1 — Supplementary Figures [file 41421_2023_547_MOESM1_ESM.pdf]

# Supplementary Fig. S1

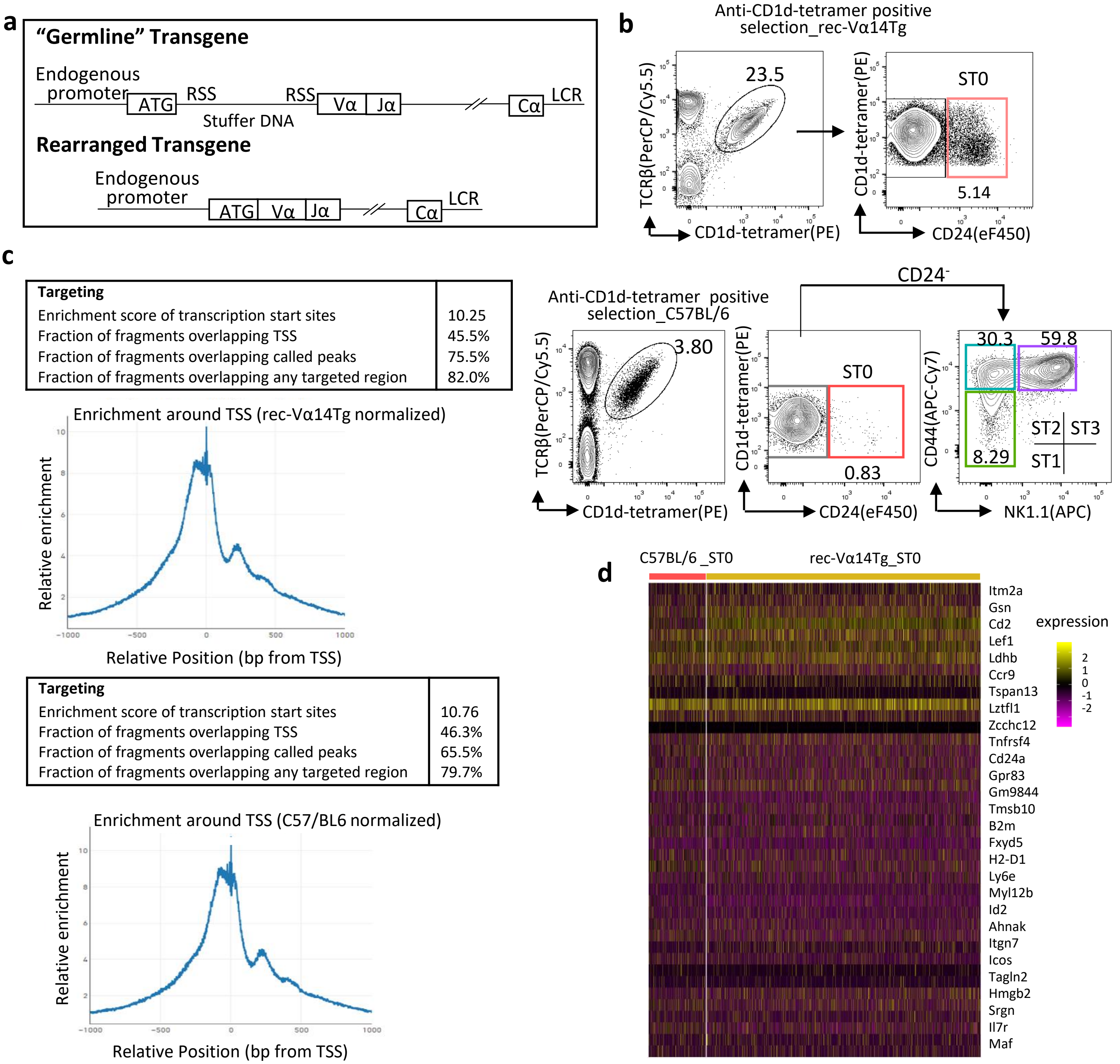


| Targeting | |
|---|---|
| Enrichment score of transcription start sites | 10.25 |
| Fraction of fragments overlapping TSS | 45.5% |
| Fraction of fragments overlapping called peaks | 75.5% |
| Fraction of fragments overlapping any targeted region | 82.0% |

| Targeting | |
|---|---|
| Enrichment score of transcription start sites | 10.76 |
| Fraction of fragments overlapping TSS | 46.3% |
| Fraction of fragments overlapping called peaks | 65.5% |
| Fraction of fragments overlapping any targeted region | 79.7% |

**Supplementary Fig. S1 Comparison of ST0 *i*NKT cell from rec-Vα14Tg mouse and C57BL/6 mouse**

a. Model for the RAG recombination dependent Vα14-Jα18 *i*NKT TCR transgene. The "germline" configuration of the transgene (top) cannot be expressed until the stuffer DNA separating the ATG from the *Vα-Jα* gene segment is removed (bottom) by RAG mediated somatic recombination. This results in near wild type timing of recombination and *TCRα* gene expression.

b. Representative flow plots of the sorting stratey used to identify ST0 ($CD24^{+}$) *i*NKT cells in rec-Vα14Tg mice (top); ST1 ($CD24^{-}CD44^{lo}NK1.1^{-}$), ST2 ($CD24^{-}CD44^{hi}NK1.1^{-}$) and ST3 ($CD24^{-}CD44^{hi}NK1.1^{+}$) in C57BL/6 mice.

c. Enrichment at transcription start sites (TSSs) for aggregated ST0 single cell profile from rec-Vα14Tg mice and C67BL/6 mice.

d. Heatmap of chromatin accessibility profiles of ST0 *i*NKT cells from C57BL/6 (red) and rec-Vα14Tg (yellow) thymus, gene labels indicate the nearest gene to each of regulator element.

# Supplementary Fig. S2

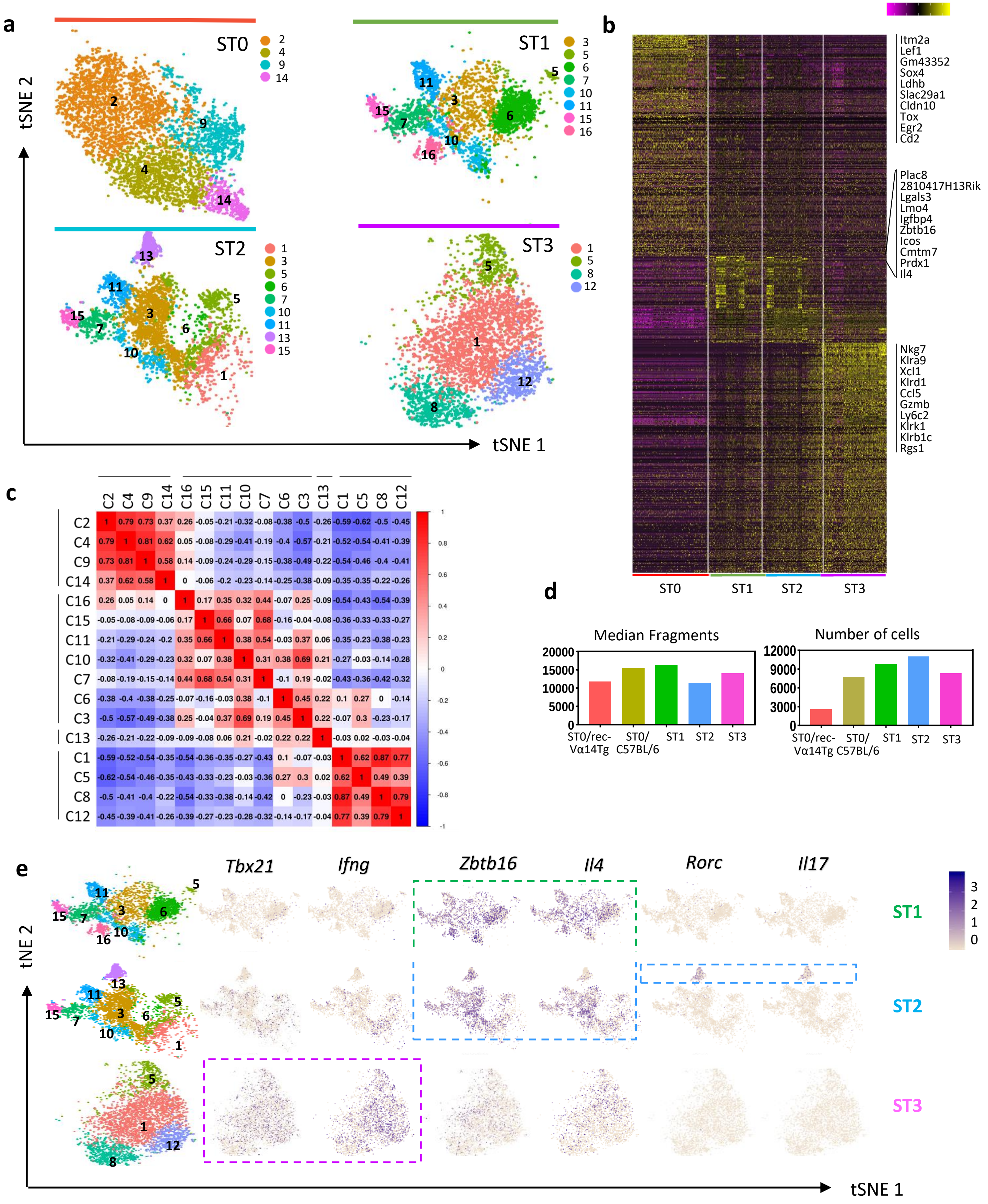

**Supplementary Fig. S2 Profiles of different stages of *i*NKT cells**

a. tSNE plots from 10X genomics single cell RNA-Seq dataset for sorted ST0, ST1, ST2, and ST3 *i*NKT cells.
b. Heatmap of the differentially regulated genes from four stages of *i*NKT cells. Each column represents gene expression for an individual cell with color coded on gene expression profiles. Top ten most different expressed genes in ST0, ST1/2, and ST3 were highlighted in the right. Yellow is up and purple is down.
c. Correlation matrix of the average expression profiles, based upon top 30 differentially expressed genes.
d. Bar graphs represent the median fragments(left) and number of cells(right) in ST0 from C57BL/6 and rec-Vα14Tg mouse, ST1, ST2 and ST3 from C57BL/6 mouse for scATAC-seq assay.
e. Feature plots depicting single-cell gene expression of *Tbx21*, *Ifng*, *Zbtb16*, *Il4*, *Rorc* and *Il17* in indicated stages of *i*NKT cells.

# Supplementary Fig. S3

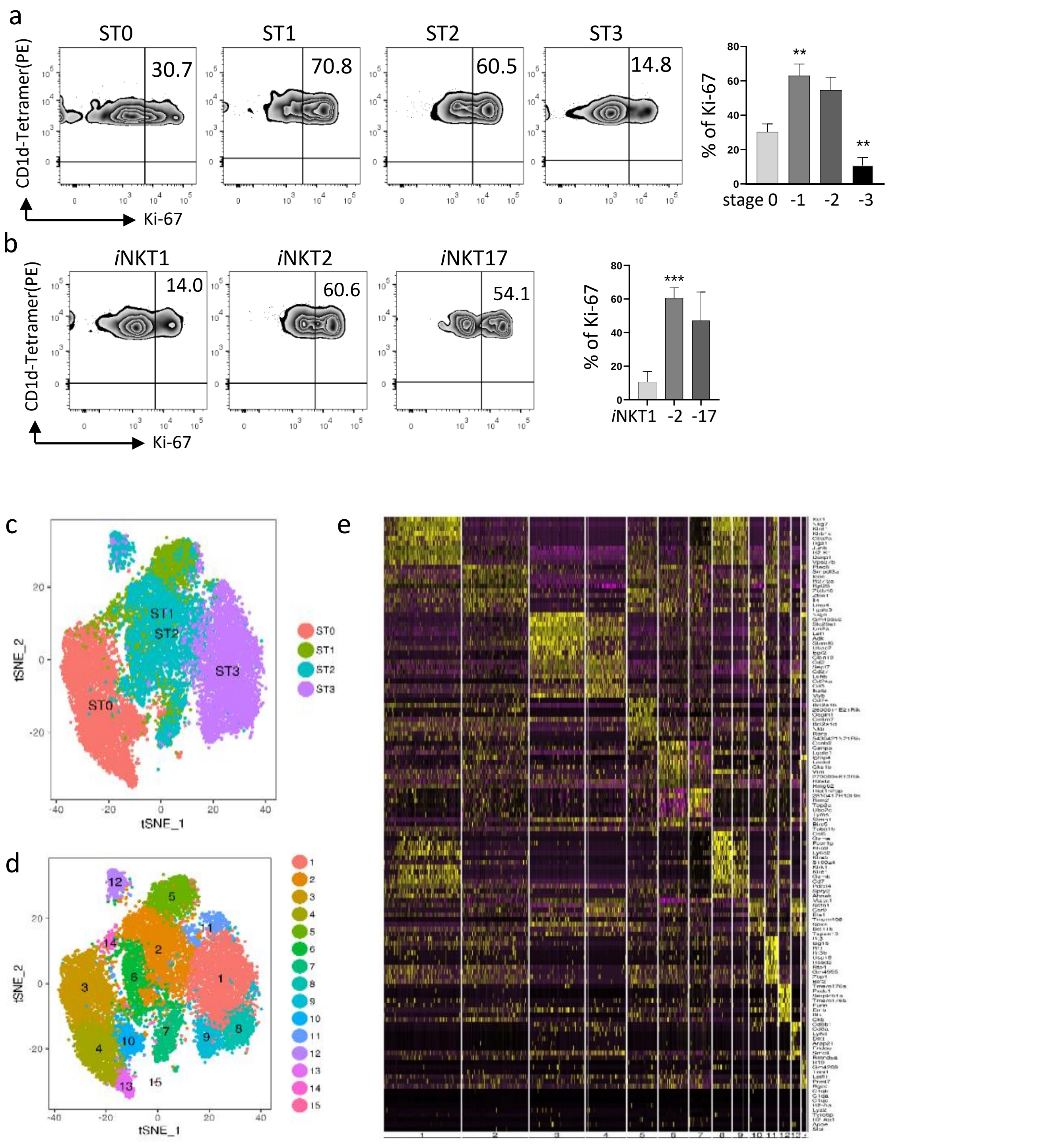


**Supplementary Fig. S3 Cluster profiles of *i*NKT cells post removing the effects of cell cycle on transcriptome**

a-b. Representative flow plots of Ki-67 expression in different stages of *i*NKT cells (a) and different subsets of *i*NKT cells (b). Data represent three independent experiments, data were analyzed by a two-sided paired t-test, ** $P<0.01$, *** $P<0.001$.

c. tSNE plots from 10X genomics single cell RNA-Seq dataset for sorted ST0, ST1, ST2 and ST3 *i*NKT cells after regressing out cell cycle effects.

d. tSNE plots from 10X genomics single cell RNA-Seq dataset for 15 clusters after regressing out cell cycle effects.

e. Heatmap of the differentially regulated genes from four stages of *i*NKT cells after regressing out cell cycle effects. Each column represents gene expression for an individual cell with color coded on gene expression profiles. Top ten most different expressed genes in each clusters were shown in the right. Yellow is up and purple is down.

# Supplementary Fig. S4

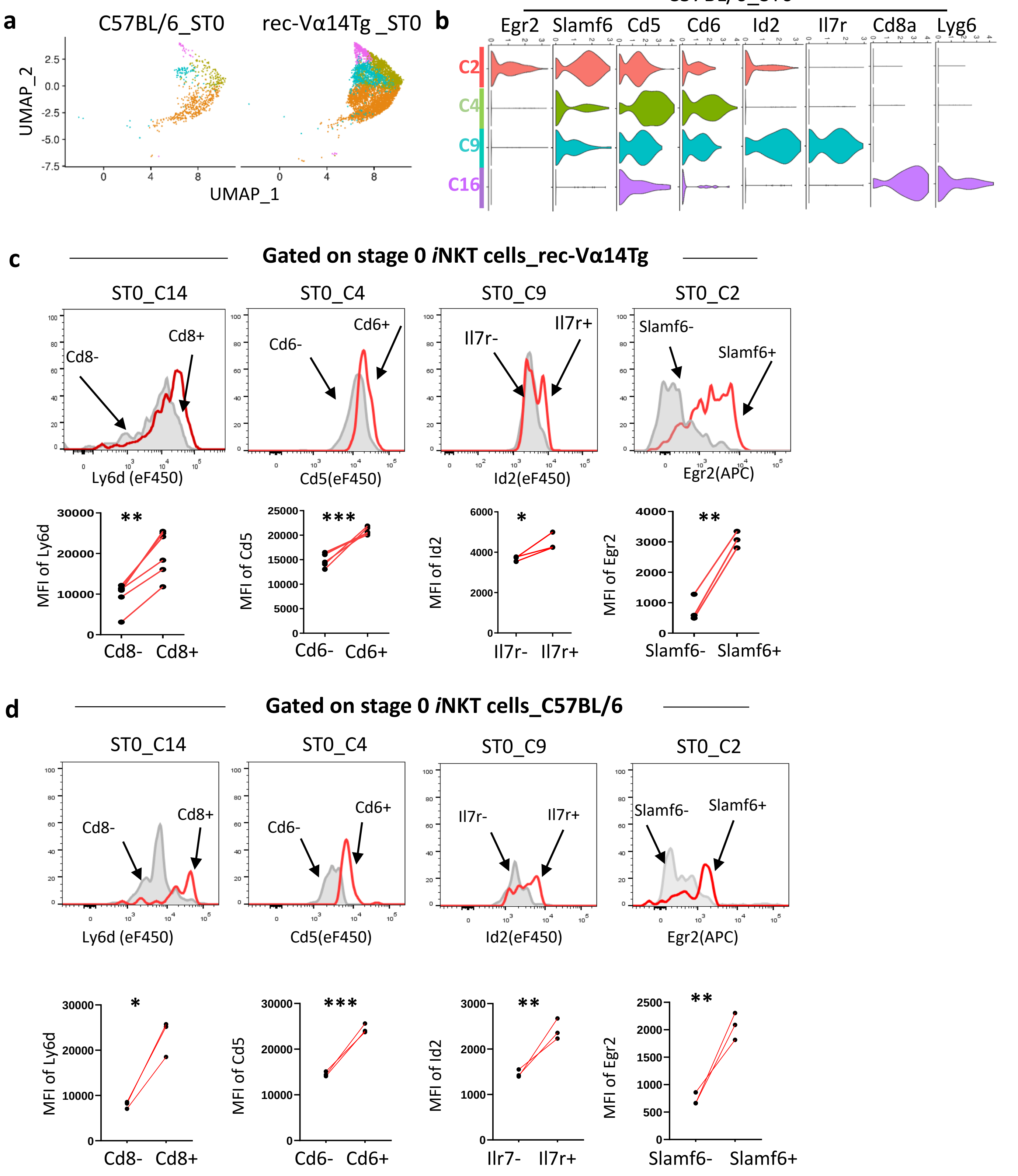


**Supplementary Fig. S4 Co-expression in stage 0 *i*NKT cells**

a. UMAP plots showing ST0 iNKT cells from C57BL/6 mouse (left) and rec-Vα14Tg mouse (right).

b. Violin plots of cluster-defining genes in each cluster derived from C57BL/6 ST0 *i*NKT cells.

c-d. Representative Ly6d expression in $Cd8^-$ and $Cd8^+$ ST0 *i*NKT cells; Cd5 expression in $Cd6^-$ and $Cd6^+$ ST0 *i*NKT cells; Id2 expression in $Il7r^-$ and $Il7r^+$ ST0 *i*NKT cells, and Egr2 expression in $Slamf6^-$ and $Slamf6^+$ ST0 *i*NKT cells from rec-Vα14Tg (c) and C57BL/6 (d) mice. Each dot represents one mouse. rec-Vα14Tg, n=5, C57BL/6, n=3. Data represent two to three independent experiments and were analyzed by a two-sided paired t-test, * $P$<0.05, ** $P$<0.01, and *** $P$<0.001.

# Supplementary Fig. S5

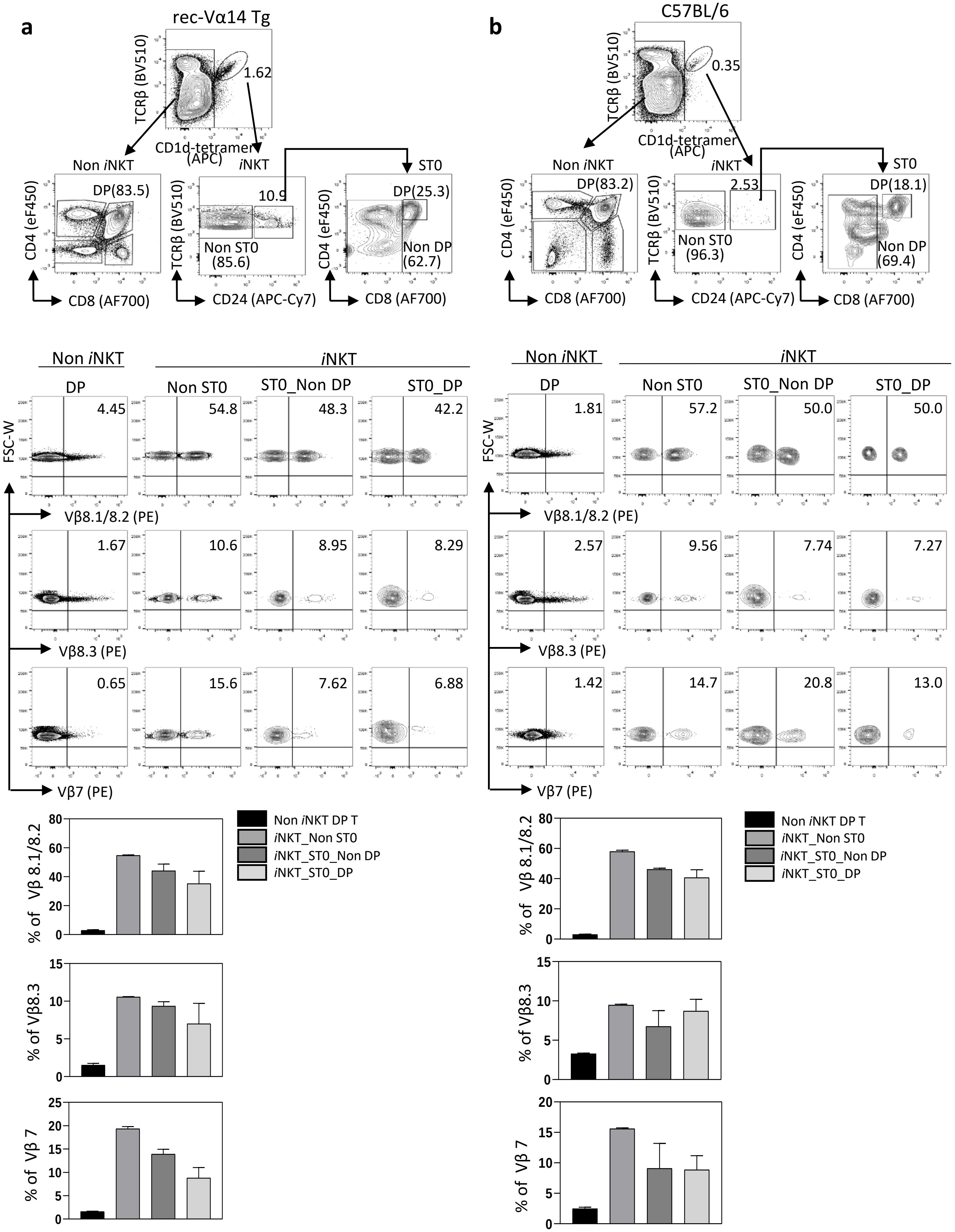


**Supplementary Fig. S5 Use of TCR Vβs in *i*NKT cells**

a-b. Representative Vβ8.1/8.2, Vβ8.3 and Vβ7 expression in indicated *i*NKT populations and non_*i*NKT DP T cells from rec-Vα14Tg (a) and C57BL/6 (b) mice. Bar graphs represent mean±s.d. n=3, data represent two independent experiments.

# Supplementary Fig. S6

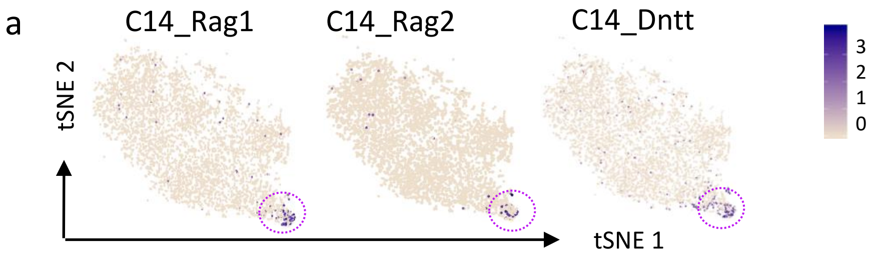


**Supplementary Fig. S6 C14 gene expression profile**

a. The same pseudotime plot as in Fig. 3a, feature plots depicting indicated genes expression trajectory in ST0 *i*NKT cell.

# Supplementary Fig. S7

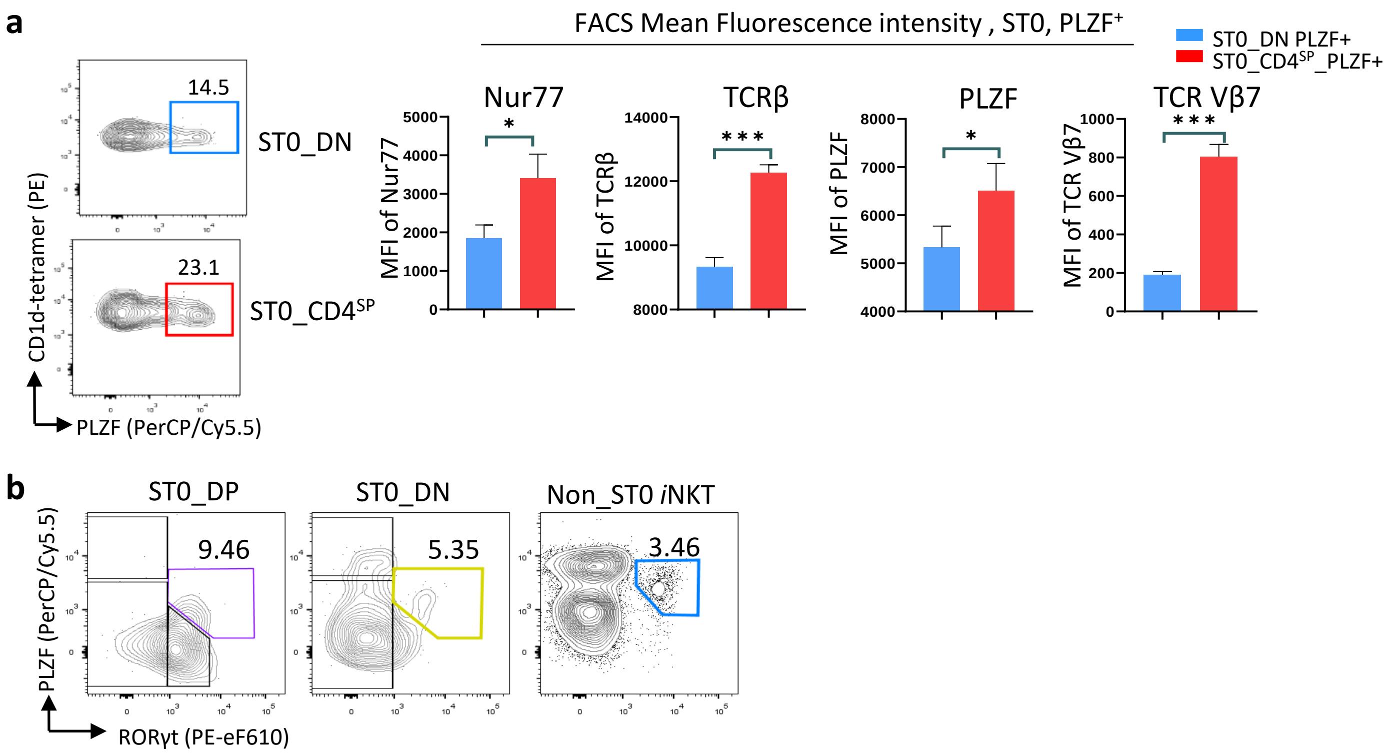


**Supplementary Fig. S7 *i*NKT lineage commitments in stage 0**

a. Representative flow plot of PLZF expression in ST0 DN and ST0 CD4$^{SP}$ (Left); Bar graphs showing the mean fluorescence intensity (MFI) of Nur77, TCRβ, PLZF, and TCR Vβ7 in PLZF positive cells from ST0 DN (Blue) and ST0 CD4$^{SP}$ (Red); mean ± s.d. n=3, data represent two independent experiments, data were analyzed by a two-sided paired t-test * $P<0.05$; *** $P<0.001$.

b. Representative flow plot of PLZF vs. RORγt expression in ST0 DP, ST0 DN and non_ST0 *i*NKT cells.

# Supplementary Fig. S8

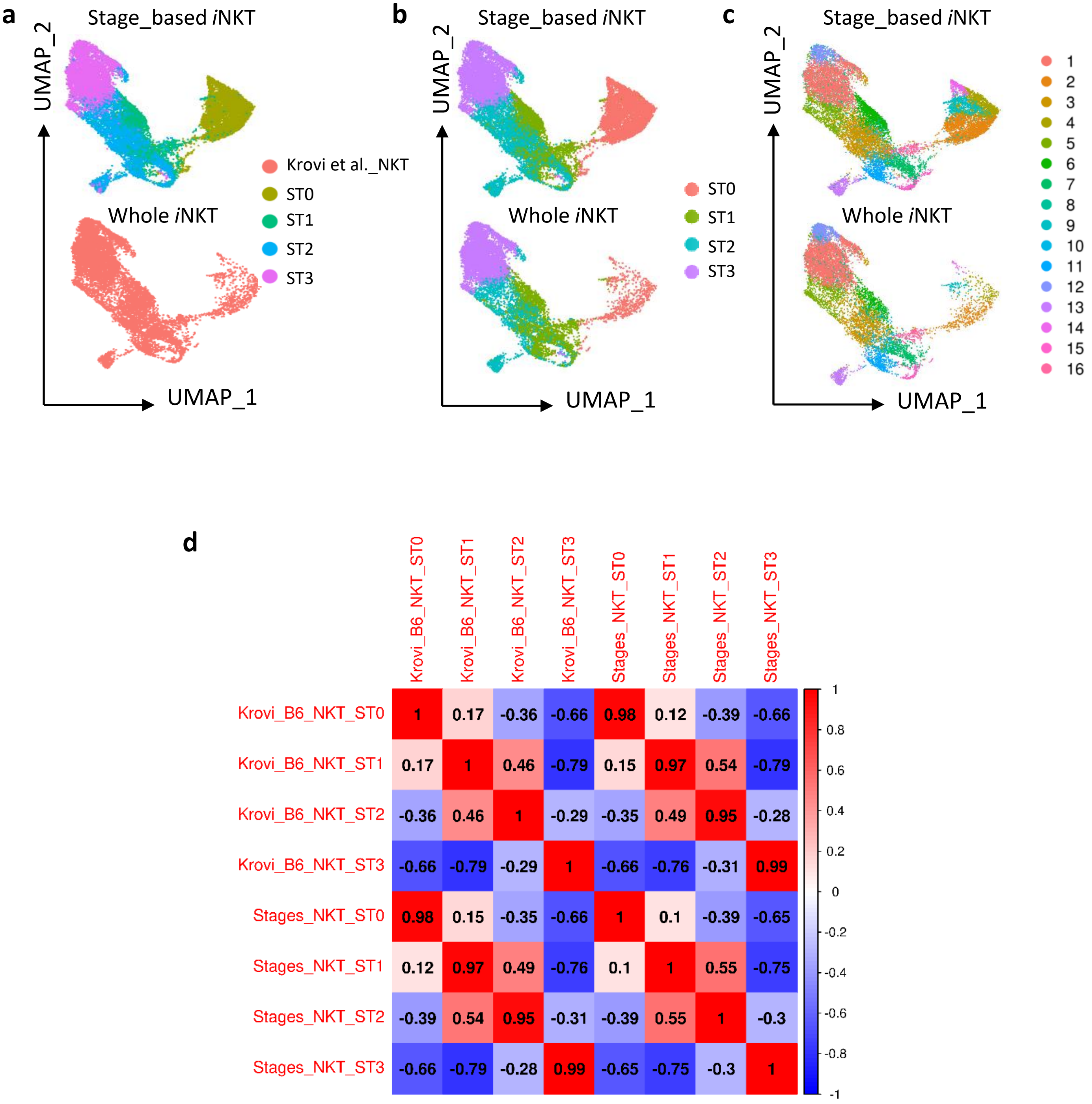


**Supplementary Fig. S8 The trajectory of *i*NKT cell development**

a. The UMAP plot, color represents different developmental stages, including ST0, ST1, ST2 and ST3 *i*NKT cells (our study, top panel), and whole *i*NKT cells (Krovi et al. study, bottom panel).
b. The UMAP plot, color represents different developmental stages, including in ST0, ST1, ST2 and ST3 *i*NKT cells (our study, top panel), and different developmental stages of *i*NKT cells from Krovi et al. study (bottom panel).
c. The UMAP plot, color represent individual *i*NKT clusters (our study, top panel), and *i*NKT clusters from Krovi et al. study (bottom panel).
d. The correlation heatmap comparing Krovi et al. dataset vs. our study for each stage based on 3,000 highly variable genes.

# Supplementary Fig. S9

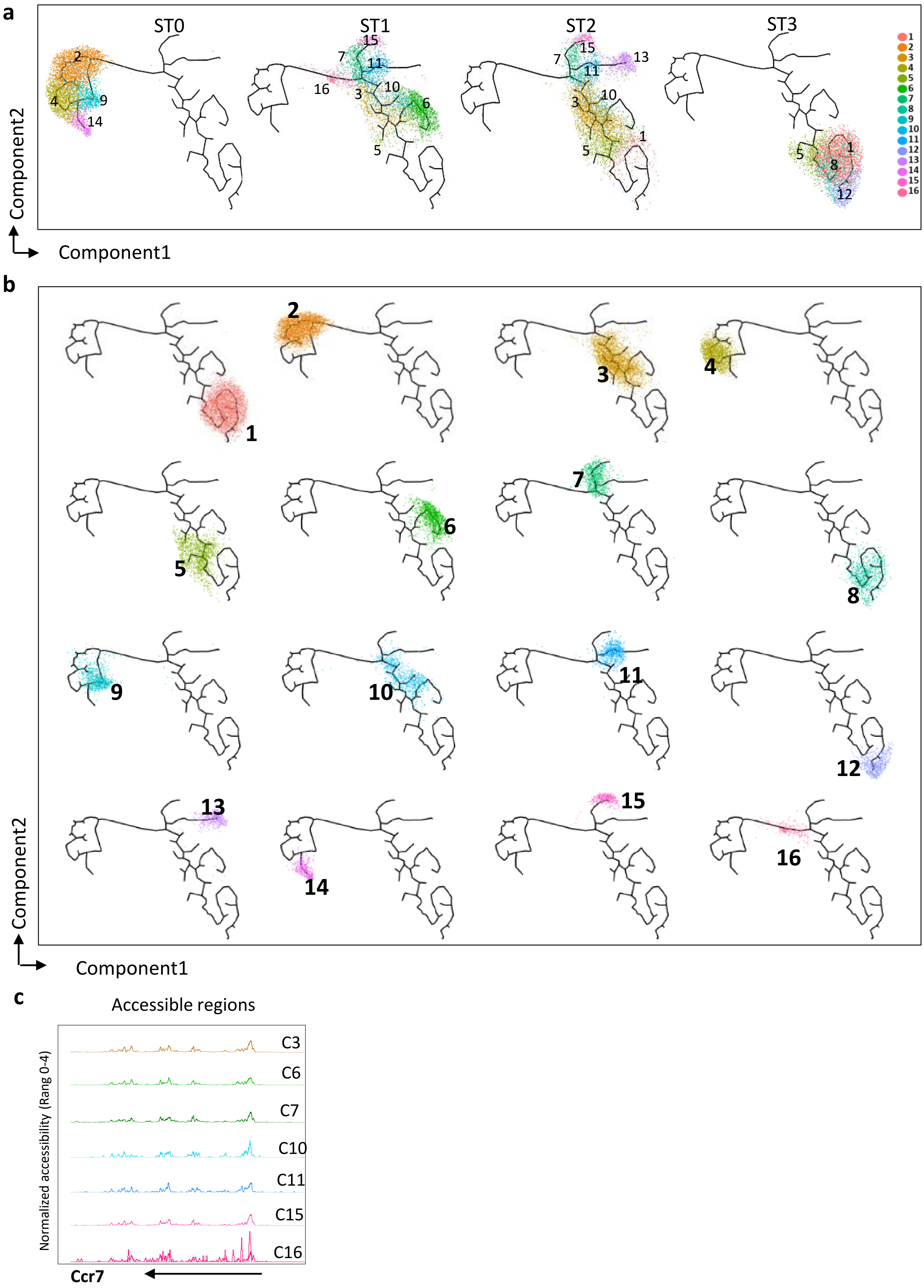

**Supplementary Fig. S9 The trajectory of *i*NKT cell development**

a. The pseudotime plot, color represents different clusters in ST0, ST1, ST2 and ST3 *i*NKT cell.
b. The pseudotime plot, color represent individual *i*NKT clusters.
c. Aggregate scATAC-seq browser tracks for *Ccr7* in ST1 *i*NKT clusters.

# Supplementary Fig. S10

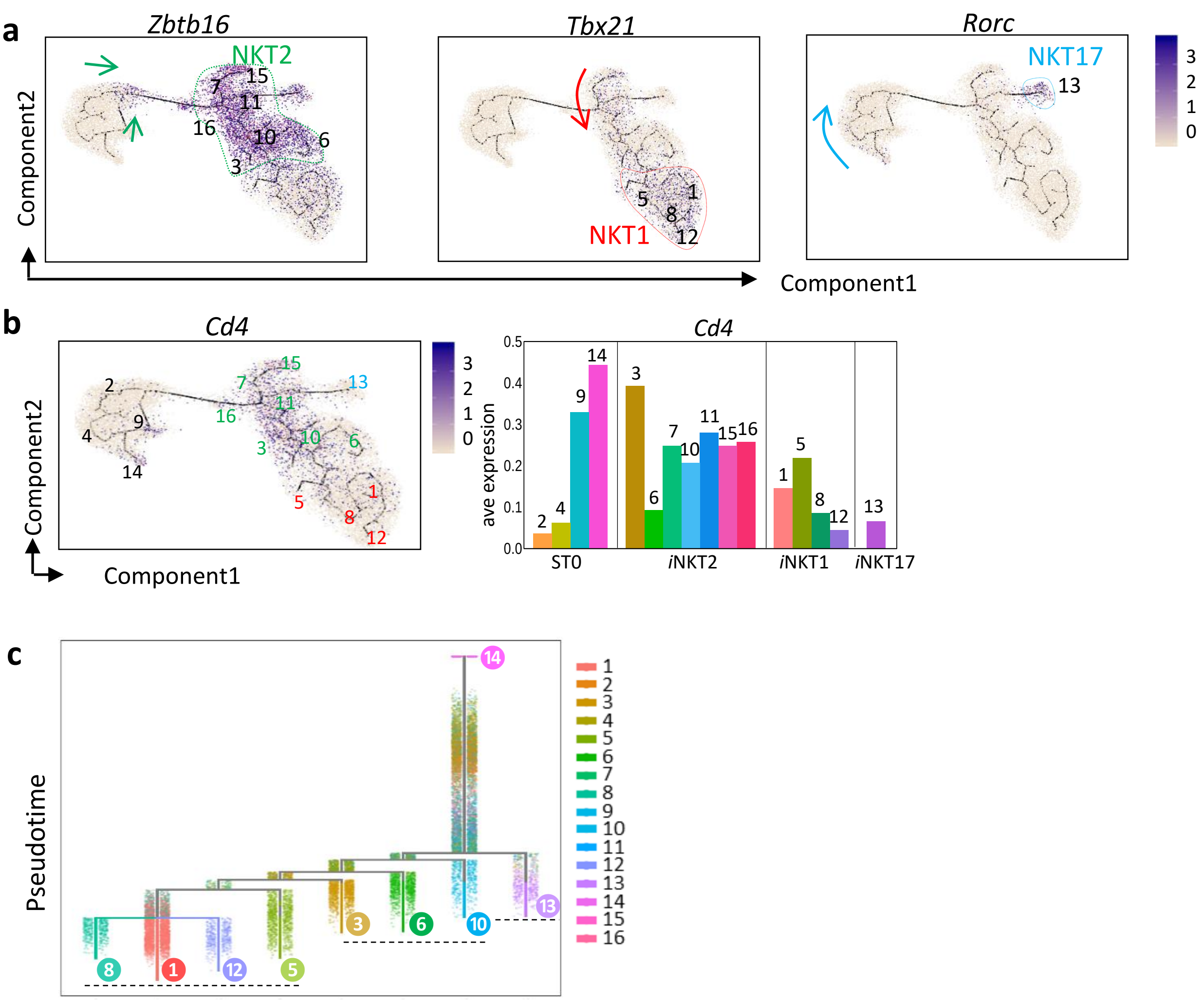


**Supplementary Fig. S10 *i*NKT cell developmental trajectory**

a. The same pseudotime plot as in Fig.4a, feature plots depicting single-cell gene expression trajectory of *Zbtb16* (left), *Tbx21* (middle) and *Rorc* (right) in the *i*NKT cell development. Narrows showing *i*NKT2, *i*NKT1 and *i*NKT17 possible differentiation directions.

b. The same pseudotime plot as in Fig. 4a, feature plots depicting single-cell gene expression trajectory of *Cd4* in *i*NKT cell development (left), bar graph represents average expression of *Cd4* in *i*NKT clusters (right).

c. URD tree plot depicts the development trajectory of *i*NKT cells, with C14 serving as root points and C8, C1,C12, C5,C3, C6, C10, and C13 identified as leaf points.

# Supplementary Fig. S11

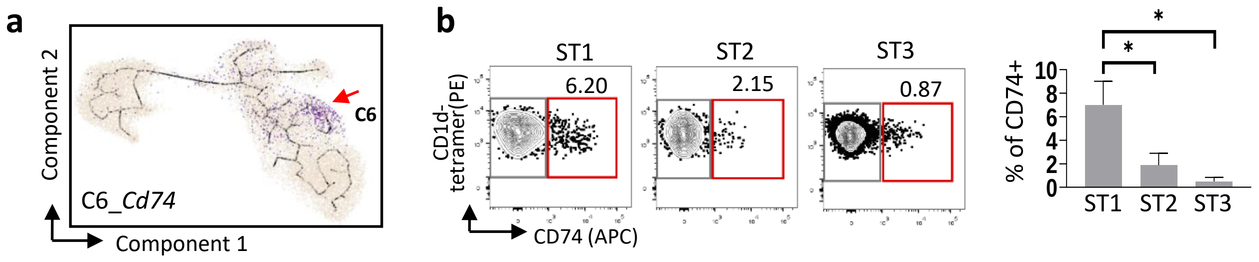


**Supplementary Fig. S11 The heterogeneity of *i*NKT2 cells**

a. The same pseudotime plot as in Fig. 4a, feature plots depicting single-cell gene expression trajectory of *Cd74* in *i*NKT cell development.

b. Representative flow plots of CD74 expression in ST1, ST2 and ST3 of thymic *i*NKT cells. Bar graphs represent mean CD74$^{+}$ *i*NKT cells ± s.d in the thymus.

# Supplementary Fig. S12

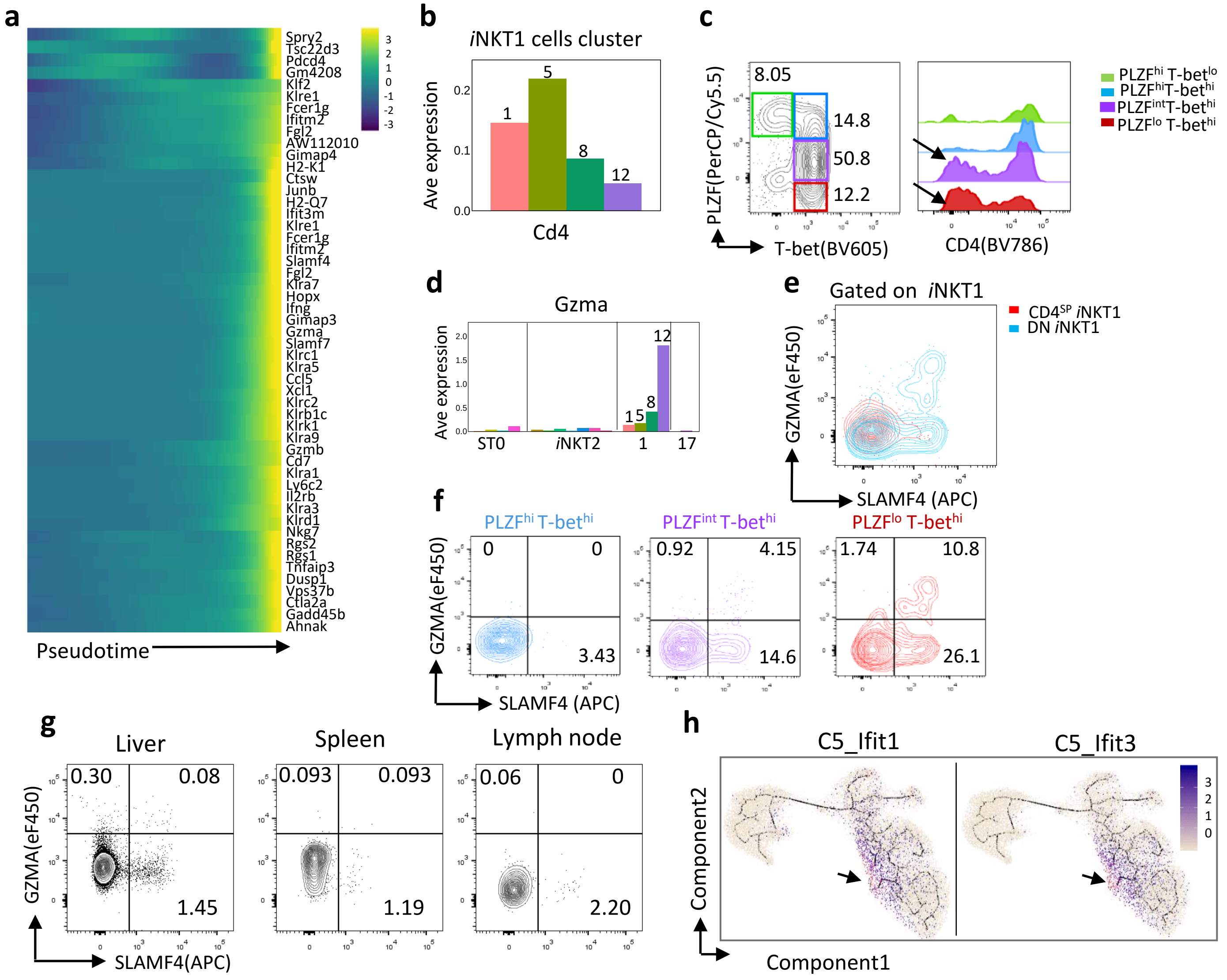


**Supplementary Fig. S12 The heterogeneity of *i*NKT1 cells**

a. Heatmap showing pseudotime ordering of top 30 genes in cluster C1, C5, C8 and C12 of scRNA-Seq data.

b. Bar graph represents average expression of *Cd4* in *i*NKT1 clusters.

c. Representative flow plot of PLZF vs. T-bet in *i*NKT cells. *i*NKT2 ($PLZF^{hi}$ $T\text{-}bet^{-}$ ) in green; *i*NKT1($PLZF^{hi}$ $T\text{-}bet^{hi}$ ) in blue; *i*NKT1($PLZF^{int}$ $T\text{-}bet^{hi}$ ) in purple and *i*NKT1($PLZF^{lo}T\text{-}bet^{hi}$) in red. Histogram showing CD4 expression in *i*NKT2 ($PLZF^{hi}$ $T\text{-}bet^{-}$) cells and different *i*NKT1 ($T\text{-}bet^{hi}$) cells, marked by PLZF high ($PLZF^{hi}$ $T\text{-}bet^{hi}$), PLZF medium ($PLZF^{int}$ $T\text{-}bet^{hi}$), and PLZF low ($PLZF^{lo}$ $T\text{-}bet^{hi}$).

d. Bar graph represents average expression of *Gzma* in *i*NKT1 clusters.

e. Representative flow plot of GZMA vs. SLAMF4 expression in gated $CD4^{SP}$ *i*NKT1 cells (red) and DN *i*NKT1 cells (blue).

f. Representative flow plots of GZMA vs. SLAMF4 expression in gated $PLZF^{hi}$*i*NKT1 cells(blue), $PLZF^{int}$*i*NKT1 cells (purple) and $PLZF^{lo}$*i*NKT1 cells (red).

g. Representative flow plots of GZMA vs. SLAMF4 expression in gated *i*NKT cells from Liver, spleen and lymph node.

h. The same pseudotime plot as in Fig. 4a , feature plots depicting single cell gene expression trajectory of *Ifit1* and *Ifit3* in *i*NKT cells development.

# Supplementary Fig. S13

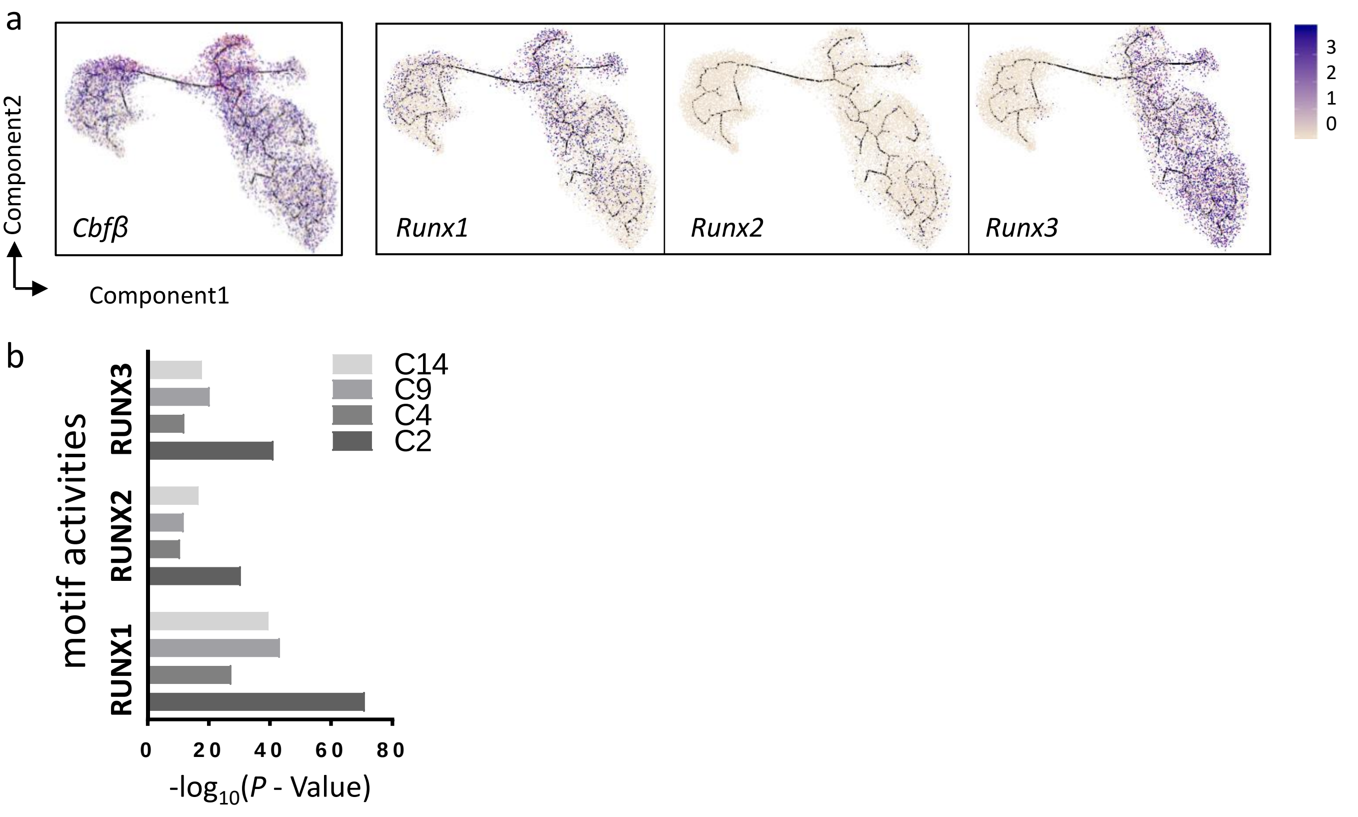


**Supplementary Fig. S13 *Runx1/2/3* expression pattern in *i*NKT cells.**

a. The same pseudotime plot as in Fig. 4a, feature plots depicting single cell *Runx1*, *Runx2* and *Runx3* expression trajectory in *i*NKT cells development.
b. Bar graph shows RUNX binding motif activities in ST0 clusters.

# Supplementary Fig. S14

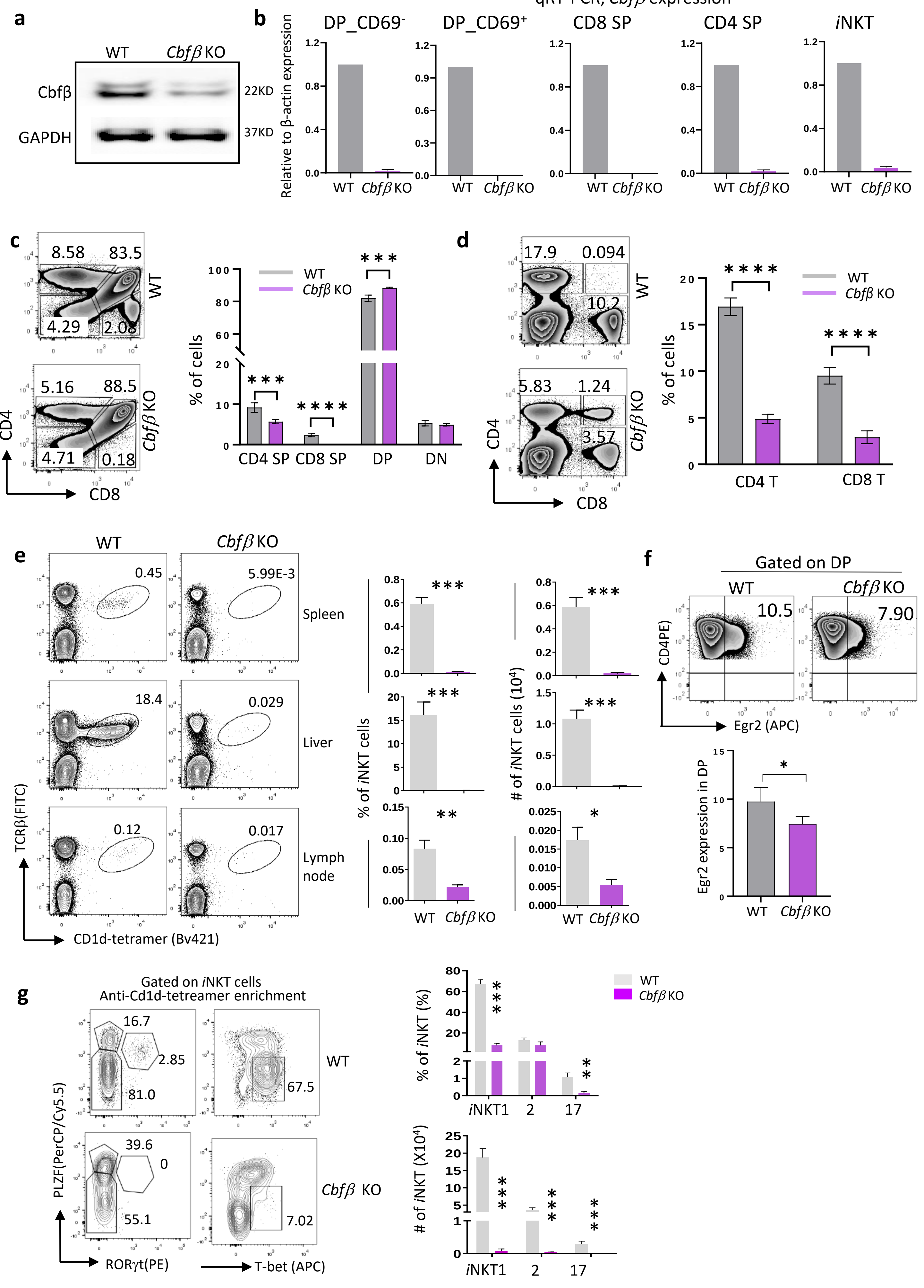

**Supplementary Fig. S14 The role of *Cbfβ* in *i*NKT cell development**

a. Western blots of Cbfβ protein expression in thymocytes from WT and *Cbfβ* KO mice. GAPDH was used as loading control.
b. qRT-PCR analysis of *Cbfβ* expression in sorted DP CD69-, DP CD69+, CD8 SP, CD4 SP and *i*NKT cells from *Cbfβ* KO and WT controls. DP, CD4+CD8+. SP, Single positive.
c. Representative flow plots of DP, DN, CD4 SP, and CD8 SP cells in the thymus from *Cbfβ* KO and WT controls (Left). Bar graph represent mean ± s.d of frequency of indicated subset of thymocytes. n=4~5, Data represent three independent experiments, data were analyzed by a two-sided non-paired t-test, *** $P$<0.001, and **** $P$<0.0001.
d. Representative flow plots of CD4 T and CD8 T cells in spleen from *Cbfβ* KO and WT controls(Left) . Bar graph represent mean ± s.d of frequency of indicated T cells (Right), n=4~5, Data represent three independent experiments, data were analyzed by a two-sided non-paired t-test, **** $P$<0.0001.
e. Representative flow plots of *i*NKT cells in spleen, liver and lymph node from *Cbfβ* KO and WT controls (Left). Bar graph represent mean ± s.d of frequency and cell number in indicated organs *i*NKT cells (Right), n=5, Data represent three independent experiments, data were analyzed by a two-sided non-paired t-test, * $P$<0.05, ** $P$<0.01, and *** $P$<0.001.
f. Representative flow plots of Egr2 expression in DP thymocyts from *Cbfβ* KO and WT controls. Bar graph represent mean ± s.d of $Egr2^{+}$ DP thymocytes in WT and *Cbfβ* KO, Data represent three independent experiments, data were analyzed by a two-sided non-paired t-test, * $P$<0.05.
g. Representative flow plots of *i*NKT1 ($PLZF^{lo}T\text{-}bet^{hi}$), *i*NKT2 ($PLZF^{hi}T\text{-}bet^{lo}$) *and* *i*NKT17 ($PLZF^{int}ROR\gamma t^{+}$) in *Cbfβ* KO and WT controls post anti-CD1d-tetramer enrichment. Bar graphs represent mean ± s.d of frequency and number of *i*NKT1, *i*NKT2, *i*NKT17 cell in *Cbfβ* KO and WT controls. n= 5, data represent three independent experiments, data were analyzed by a two-sided non-paired t-test, * $P$<0.05, ** $P$<0.01, and *** $P$<0.001.

# Supplementary Fig. S15

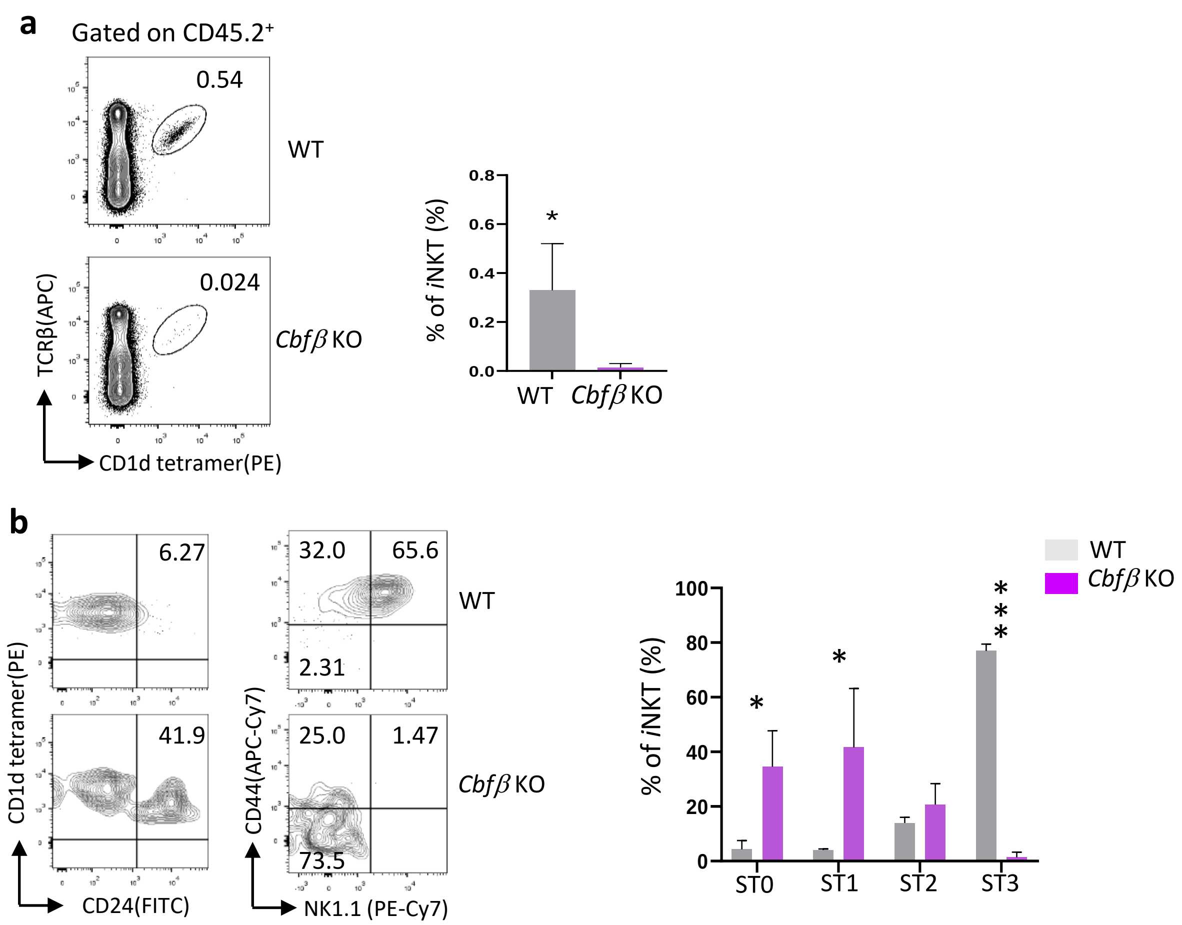


**Supplementary Fig. S15 The role of *Cbfβ* in *i*NKT cell development in bone marrow chimera transfer model.**

a. Representative flow plots of *i*NKT cells from *Cbfβ* KO and WT bone marrow (left). Bar graph represents mean± s.d of *i*NKT cell frequency derived from *Cbfβ* KO and WT bone marrow. WT bone marrow donor, n=3. *Cbfβ* KO bone marrow donor, n=4; data represent two independent experiments, data were analyzed by a two-sided non-paired t-test * $P<0.05$.

b. Representative flow plots of different stages of *i*NKT cell derived from *Cbfβ* KO and WT bone marrow (left). Bar graph represents mean± s.d of *i*NKT cell frequency at different stages. WT bone marrow donor, n=3. *Cbfβ* KO bone marrow donor, n=4; data represent two independent experiments, data were analyzed by a two-sided non-paired t-test * $P<0.05$; *** $P<0.001$.
